# Multi‐Scale Structural Effects of External Electrical Fields of Melt Tracks in Laser Powder Bed Fusion

**DOI:** 10.1002/advs.202518344

**Published:** 2026-02-08

**Authors:** Ankit Das, Shuichiro Hayashi, Craig B. Arnold

**Affiliations:** ^1^ Department of Mechanical and Aerospace Engineering Princeton University Princeton New Jersey USA; ^2^ Princeton Materials Institute Princeton University Princeton New Jersey USA

**Keywords:** bimodal laser processing (BLP), electric field (EF), powder bed fusion (PBF), structural refinement, unimodal laser processing (ULP)

## Abstract

The dynamics of the melt pool critically influence the structure and resultant properties of resolidified metals in laser‐based powder bed fusion and related 3D printed applications. While the influence of external electric fields on the surface and bulk properties of fluids at ambient conditions is well documented, their impact on the transient, high‐temperature melt pools characteristic of powder bed fusion remains largely unexplored, and their broader applicability is unclear across multiple length scales. Here, we reveal how external non‐contact EFs influence both macro‐ and microstructures during laser scanning. EFs have the ability to influence structure through a fundamentally different mechanism than thermal approaches. Qualitative and quantitative analyses show that EFs drastically improve continuity and stability of metal tracks while enhancing surface smoothness in micro‐ and nano‐scale. Beyond the surface, EFs drive the formation of equiaxed grains, promoting grain refinement in bulk. Structural effects depend on EF type as well as the orientation and direction with respect to the laser scanning direction. Moreover, we demonstrate that EFs can be effectively coupled with advanced beam‐shaping strategies, yielding synergistic structural control and indicating their potential as a versatile and adaptable tool for next‐generation advanced manufacturing systems.

## Graphical abstract

Laser material processing is a promising technique for fabricating metal structures. Here, external electric field (EFs) assisted laser processing of metallic powders are investigated. EFs affect the melt tracks through changes in their stability and influence the grain structure in conjunction with laser intensity distribution methods. The EF‐assisted structures have shown enhanced surface smoothness across multiple length scales.

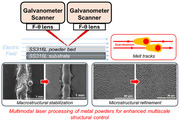

## 1 Introduction

Thermophysical properties and their associated phenomena critically influence the structural features of metals upon resolidification. By tuning parameters such as peak temperature, heated volume, and cooling rate, characteristics such as overall macro‐scale morphology, surface quality, and micro‐scale grain size can be controlled. However, alterations in these parameters often lead to cascading effects on other aspects of the process, limiting flexibility and precision. This challenge is particularly pronounced in laser‐based metal advanced manufacturing (AM), which relies on high‐energy lasers to locally melt metallic powders. While laser‐based AM offers exceptional precision, enabling the selective repairing of damaged components and manufacturing of intricate 3D architectures [1, 2], this same precision gives rise to steep thermal gradients that induce melt pool instabilities. These instabilities often result in macro‐scale discontinuities [3, 4, 5, 6, 7] and undesirable grain structures at the micro‐scale [8], compromising process resolution [9] and structural reliability [2, 10].

External stimuli, such as magnetic [11], acoustic [12], and electric stimuli [13, 14] have garnered interest for their ability to influence metal resolidification independently of thermal conditions. Among electric stimuli, electric fields (EFs) are particularly promising as they can modulate surface tension‐driven behaviors [15, 16], giving rise to effects like electrowetting, fluid displacement, and capillary modulation [16, 17]. Despite this potential, EF integration in laser‐based AM has been limited, primarily because conventional EF setups rely on contact‐mode configurations, where an electrode must physically touch the liquid metal, a setup incompatible with typical systems. Demonstrations of non‐contact EF application have shown that molten metals can indeed be manipulated during laser processing, leading to altered resolidified geometries. However, such studies have thus far been restricted to localized single‐spot melting of powder beds, effectively 0D systems. To extend applicability to a broader context of laser processing, it is critical to investigate EF effects under laser scanning, or higher dimensional regimes. Moreover, while prior work focused on macroscopic geometrical deformations, the underlying microstructural effects under an EF during resolidification remain poorly understood.

Here, we reveal how external non‐contact EFs influence the structural features of resolidified laser‐scanned metal tracks across multiple length scales. Both static (DC) and time‐varying (AC) EFs improved track continuity and stability at the macroscale, while surface evaluations revealed enhanced smoothness of the surface at micro‐ and nanoscales. Beyond geometrical and surface aspects, in the bulk, EF application drives the formation of equiaxed grains and promotes grain refinement. Furthermore, combining EFs with advanced beam‐shaping strategies leads to synergetic effects, achieving close to full equiaxed, fine and low‐aspect‐ratio grains while retaining improved macroscopic features. This study lays the necessary groundwork to show the broader applicability of EFs in future multi‐dimensional AM systems for dynamic adaptive control.

## 2 Results and Discussions

### 2.1 Structural Effects of EF During Conventional Laser Scanning

**FIGURE 1:**
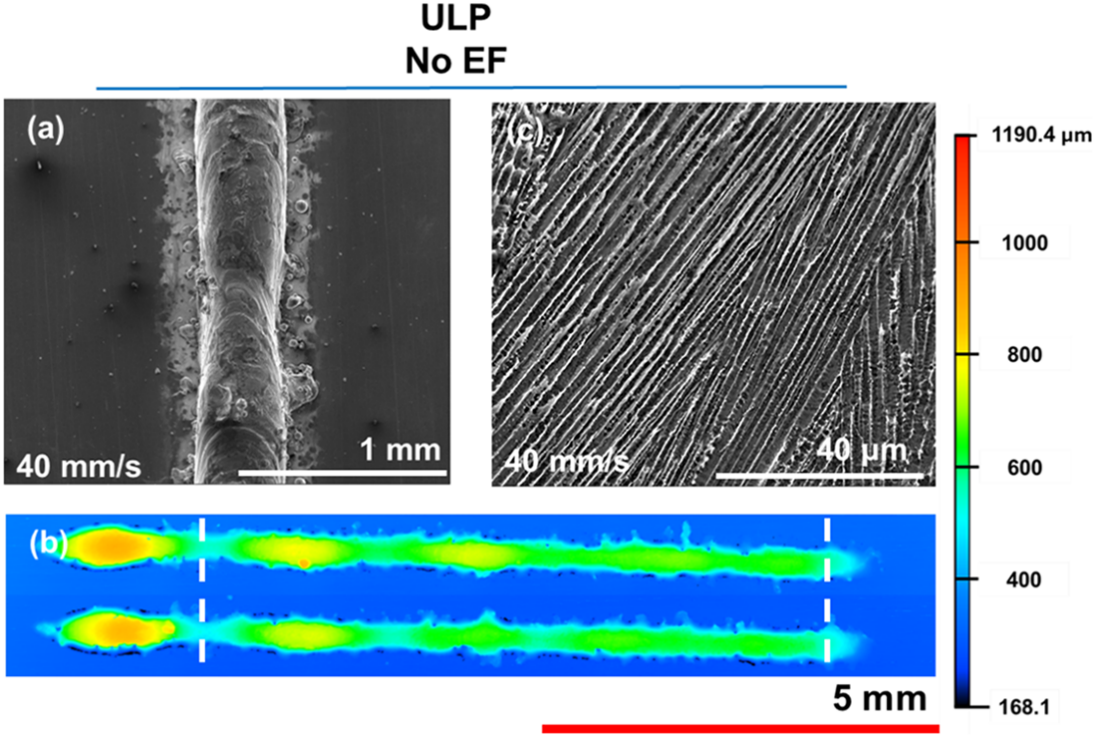
Top‐view (a) SEM and (b) confocal images of the melt track. Two tracks are shown in (b) to represent repeatability, and were fabricated by the same parameters. Color scale on the right corresponds to the height profile for confocal mapping. The quantitative analysis was conducted over the length between the two white dashed lines. (c) Cross‐sectional SEM image of the melt track revealing the microstructure. All tracks shown were prepared with a laser power and scanning speed of 200 W and 40 mm/s, respectively.

Figure 1 shows a single line metal track formed by 1D scanning of a conventional Gaussian laser beam (unimodal laser processing, ULP) across a powder bed, without an applied EF. The rationale for the selected laser parameters is provided in the Materials and Methods section and (Figures S1 and S2). Scanning electron microscope (SEM) image reveals a locally continuous track with only minor necking or balling, confirming the absence of major morphological discontinuities (Figure 1a). Moreover, the confocal images show slight long‐range height variations along the track, and the presence of little undulations arising from Rayleigh instabilities (Figure 1b). The large hump observed at the beginning of the scan is a common artifact of laser processing [18, 19], which is ignored, and only a section of the melt tracks are analyzed, as shown in Figure 1b as the length between the two white dashed lines. This commonly observed phenomenon is due to longer irradiation times and the resulting larger melt volumes obtained at the start of the laser scan. Cross‐sectional analysis (Figure 1c) revealed the predominance of columnar grains, consistent with conventional ULP of SS316L, where steep thermal gradients and high heat inputs drive columnar growth [20]. Such grains are undesirable in AM parts due to poor mechanical performance and crack susceptibility [21].

**FIGURE 2:**
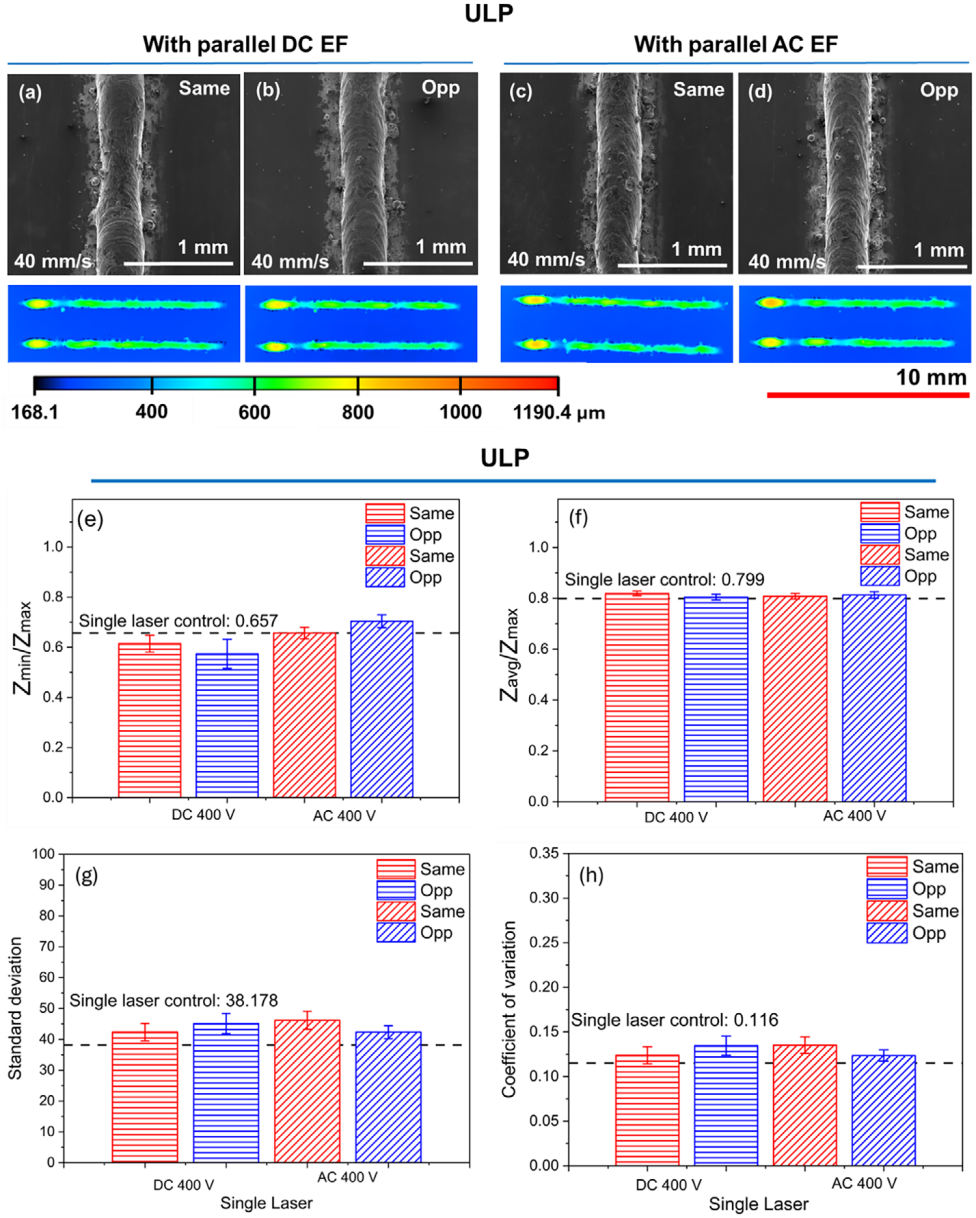
Top‐view SEM and confocal images of melt tracks fabricated under (a) same and (b) opposite parallel orientation DC EF (400 V), and (c) same and (d) opposite parallel orientation AC EF (400 V, 6 kHz). Color scale corresponds to normalized height profile for all confocal mappings. (e) $\left(\frac{Z_{min}}{Z_{max}}\right)$, (f) $\left(\frac{Z_{avg}}{Z_{max}}\right)$, (g) standard deviation, and (h) coefficient of variation assessment parameter values of melt tracks fabricated with different parameters.

Figure 2 compares melt tracks fabricated under identical laser parameters with either a static (400 V DC) or alternating (400 V AC at 6 kHz) EF applied parallel to the laser scan. The applied potential (V) can be converted into the applied electric field strength (Vm^−1^) across the electrodes by simply multiplying by a factor ∼50 (details in Experimental Section). Therefore, an applied potential of 400 V corresponds to an electric field strength of 20 × 10^3^ Vm^−1^. The applied EF parameters were set according to prior work. Two parallel orientations were used, with the EF aligned either in the same or opposite direction to the laser scan. For both parallel DC and AC EFs, the tracks remained continuous and relatively stable regardless of the orientation (Figure 2a–d), similar to those without an EF (Figure 1a,b). To quantify these observations, track continuity and stability were assessed using height‐based assessment parameters. Figure 2e shows a coupled metric for continuity and undulations, or the ratio of minimum to maximum track height $\left(\frac{Z_{min}}{Z_{max}}\right)$, for EF conditions shown in Figure 2a–d. Values closer to 0 indicate the presence of a track discontinuities due to large undulations. $\left(\frac{Z_{min}}{Z_{max}}\right)$ remained comparable between control and EF‐assisted tracks, both DC and AC, indicating no significant differences in continuity. Similar inference was drawn by the average‐to‐maximum height ratio $\left(\frac{Z_{avg}}{Z_{max}}\right)$ (Figure 2f). By contrast, localized stability metrics, or the standard deviation and coefficient of variation, revealed subtle differences (Figure 2g,h). This suggests that while continuity is preserved the EF introduces small surface undulations.

**FIGURE 3:**
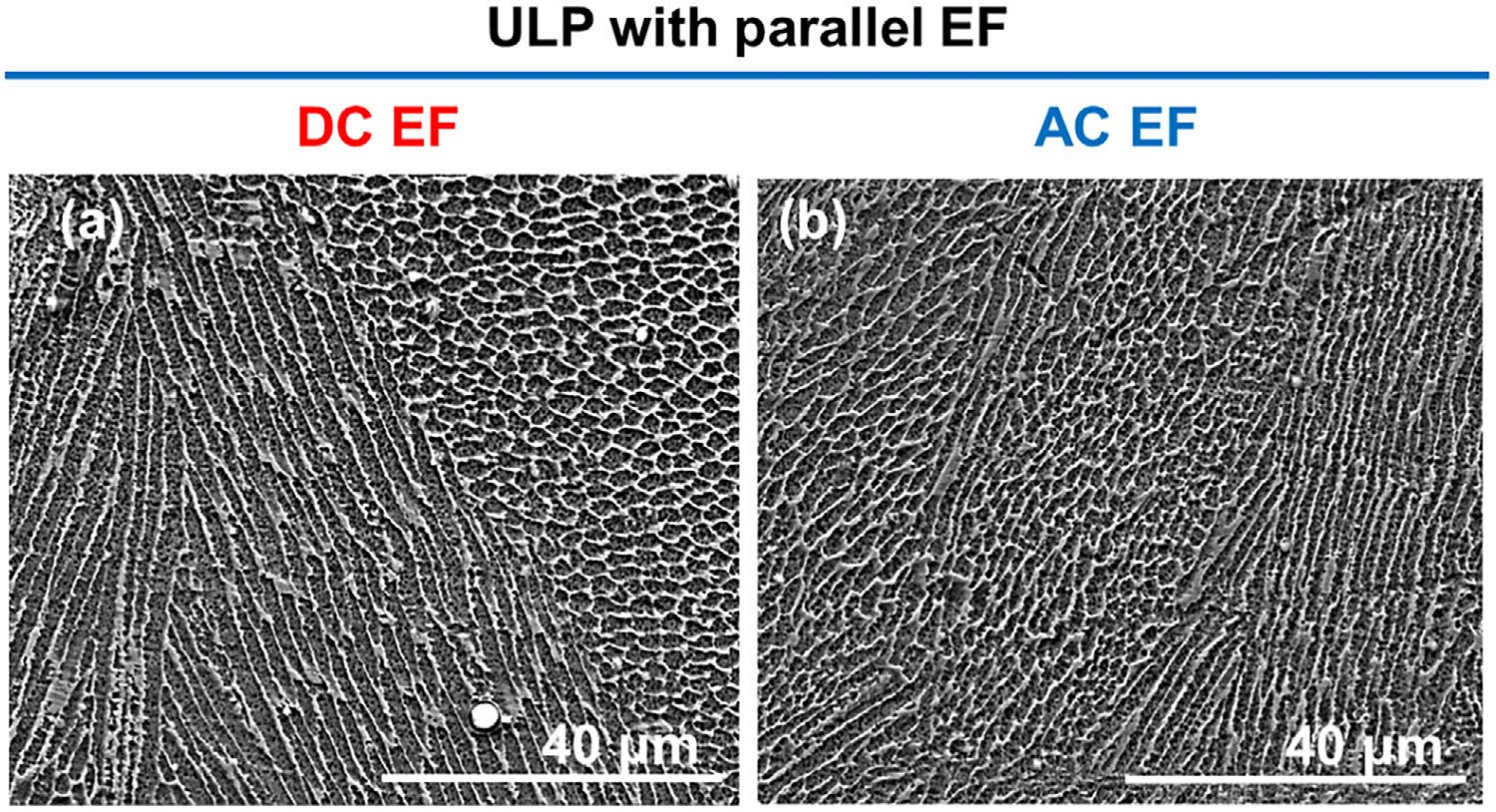
Microstructures formed under a parallel (a) DC (400 V) and (b) AC (400 V, 6 kHz) EF.

On the other hand, microstructural analysis revealed clear visual differences in grain morphology with EF application. Microstructures for the “same” orientated EF is reported, as there was little to no influence of EF orientation on microstructure. While ULP tracks were dominated by columnar grains (Figure 1c, the application of either DC or AC EFs promoted the formation of equiaxed grains (Figure 3a,b). Although columnar regions persisted, the increased presence of equiaxed structures demonstrates that EFs can actively influence solidification pathways. Based on thermal parameters and process conditions, the presence of columnar or/and equiaxed cells has been reported in the past and is typical for SS316L [22, 23]. Typically, when microstructures are similar in nature (type and size), it indicates that the cooling rates are similar at this location, particularly at the melt center [22]. In this work, considering the analyzed location, the microstructure should have been closely related. However, the different nature of the microstructure indicates that EFs affect the microstructure, and a mechanism beyond the thermal control is involved.

### 2.2 Interaction of EFs with Advanced Beam‐Shaping

**FIGURE 4:**
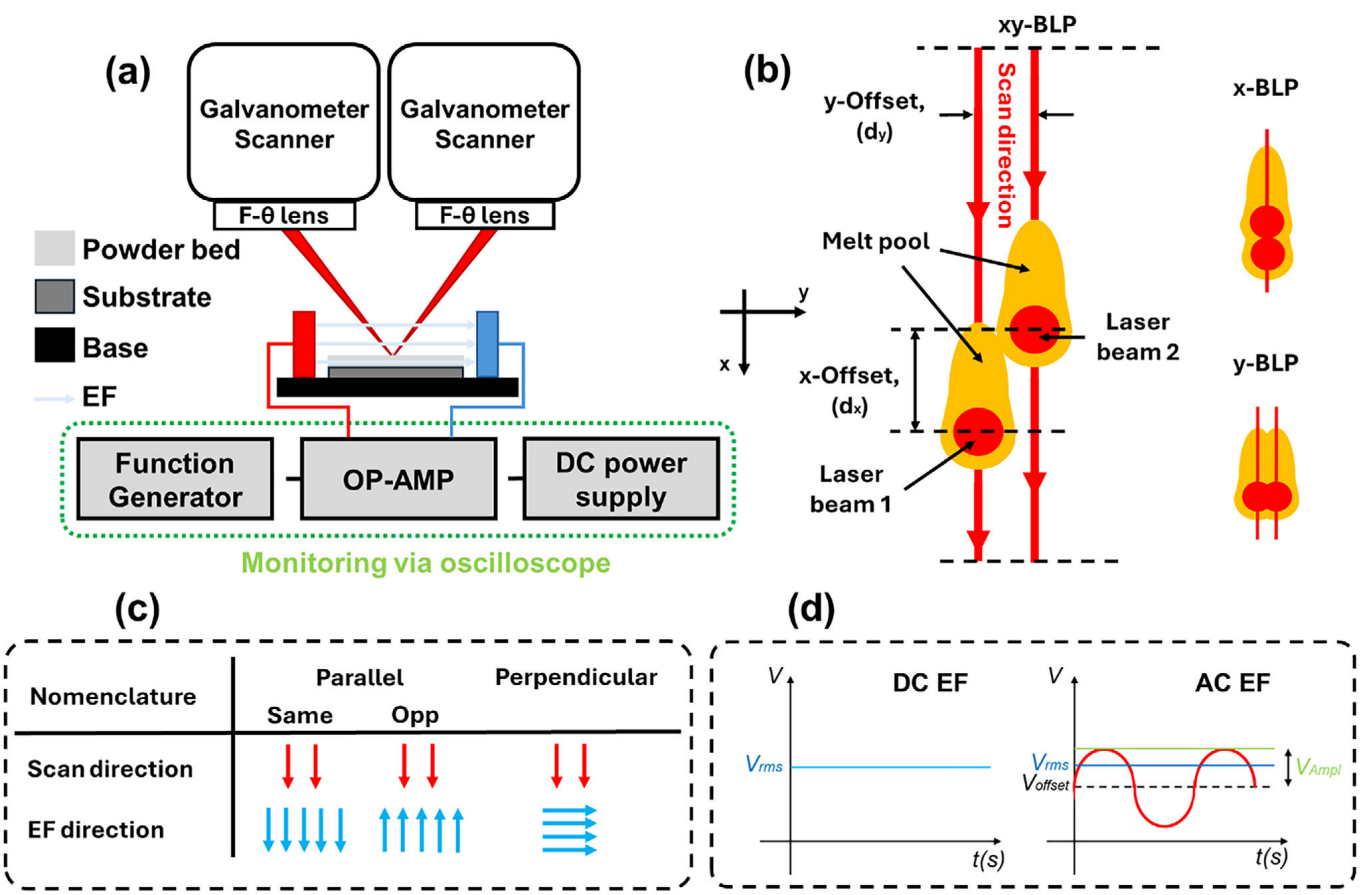
(a) Schematic of the experimental setup, (b) BLP configuration, (c) nomenclature for experiments, and (d) applied EF waveforms.

In addition to the ULP technique and its effects on the addition of EFs, investigations were made to observe the effect of EFs when integrated with beam‐shaping strategies. Considering past beam shaping strategies based on redistribution of the laser energy [24, 25], two independent lasers were superimposed at different spatial offsets [24, 26, 27], overall representing bimodal laser processing (BLP) (Figure 4a,b). Furthermore, similar to ULP, differently orientated EFs (Figure 4c,d) were applied to observe its effects on BLP macrostructure and microstructure.

**FIGURE 5:**
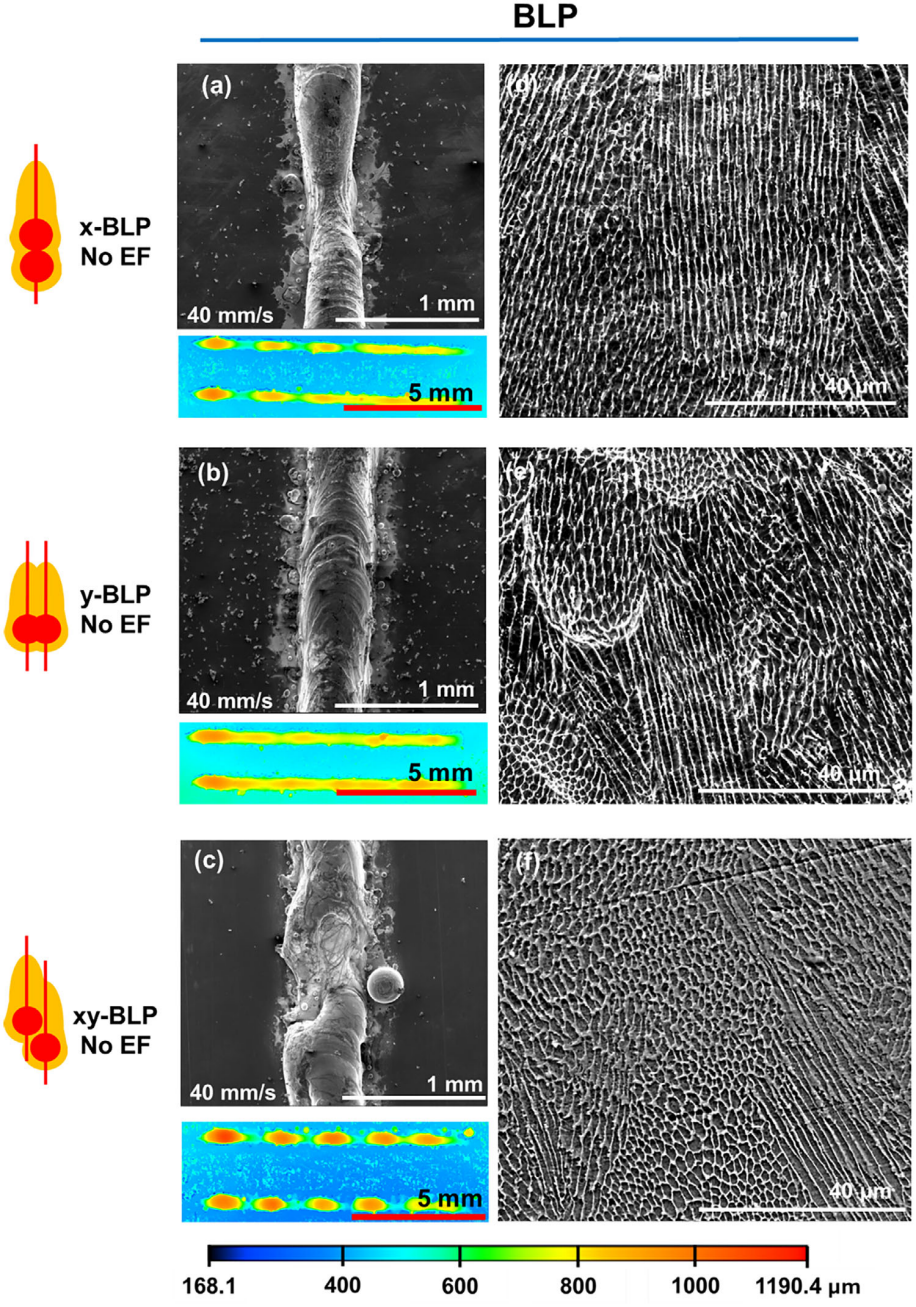
SEM and confocal images of melt tracks fabricated by (a) x‐BLP, (b) y‐BLP, and (c) xy‐BLP, without EF application. Cross‐sectional SEMs of resulting microstructures for (d) x‐BLP, (e) y‐BLP, and (f) xy‐BLP. Color scale corresponds to the normalized height profile for all confocal mappings.

Figure 5 shows SEM images of the melt tracks formed by BLP without an EF. Three spatial configurations relative to the *x*‐axis scan direction were tested. In x‐BLP, or offset only along x, the tracks were continuous (Figure 5a) but exhibited slight Rayleigh‐derived humping (Figure 5 confocal; Figure S4) [28], with track widths comparable to ULP under identical power and speed. In y‐BLP, or offset only along y, the tracks were also continuous (Figure 5b) but more stable with less undulations (Figure 5b confocal). In xy‐BLP, or offset along both x and y, severe track discontinuities (Figure 5c) and periodic instabilities (Figure 5c confocal) were observed, attributed to coalescence phenomena [29]. The y and xy‐BLP melt tracks both exhibited a ∼100 µm increase in width compared to ULP, comparable with the ∼110 µm y‐offset expanding the meltpool.

Even without EF application, BLP leads to distinct microstructural changes compared to ULP. In x‐BLP, effectively creating a melt pool elongated parallel to the direction of laser scanning, partial formation of equiaxed grains was observed (Figure 5d) relative to the predominantly columnar grains of ULP (Figure 1b). In y‐BLP, effectively creating a melt pool elongated perpendicular to the direction of laser scanning, the proportion of equiaxed grains increased further (Figure 5e). These observations reflect changes in temperature gradients and melt flows due to geometrical alternation from the conventional circular melt pool of ULP [25]. On the other hand, xy‐BLP shows the highest proportion of equiaxed grains among all configurations as seen from Figure 5f, owing to combined optimized effect of temperature gradients and melt pool dynamics during resolidification [24].

**FIGURE 6:**
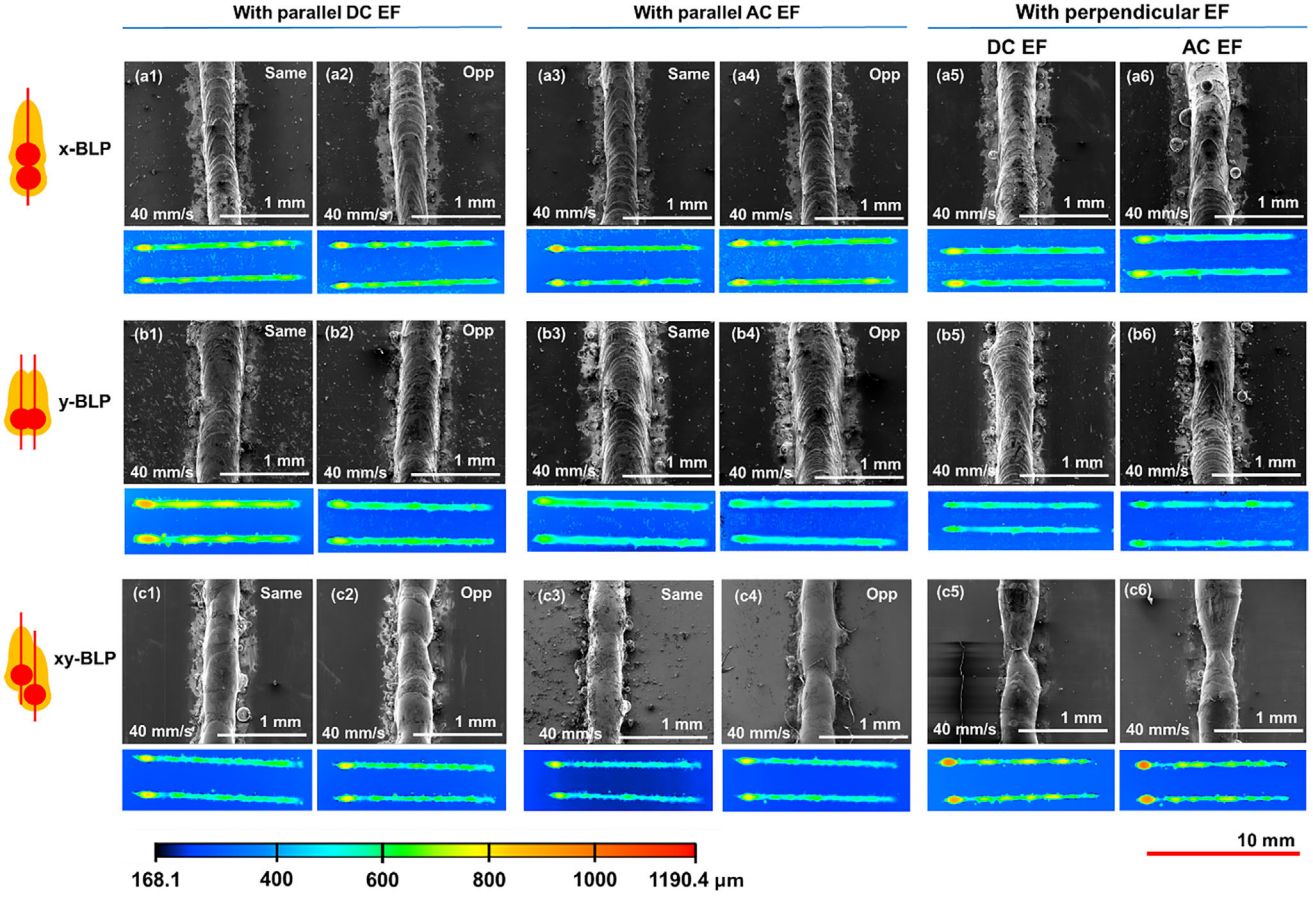
SEM and confocal images of melt tracks fabricated by (a) x‐BLP, (b) y‐BLP, and (c) xy‐BLP, under (1) parallel same DC, (2) parallel opposite DC, (3) parallel same AC, (4) parallel opposite AC, (5) perpendicular DC, and (6) perpendicular AC EFs. Color scale corresponds to the normalized height profile for all confocal mappings. Scale bar on bottom right corresponds to the scale for all confocal images.

Upon application of a parallel EF, the continuity and stability of the melt tracks for x‐BLP (Figure 6a) and y‐BLP (Figure 6b) were preserved regardless of the orientation (same vs opposite) and type (DC vs AC) of EF. Whereas the discontinuous metal tracks in xy‐BLP (Figure 5c), became visibly continuous and stabilized under the influence of both parallel DC and AC EFs (Figure 6c1–c4).

Considering the interdependency between the direction of the EF and the scan direction of BLP, the effects of perpendicular EFs were investigated. For x‐BLP, perpendicular EFs lead to negligible changes in macrostructural continuity compared to its parallel counterparts (Figure 6). Tracks appeared slightly wider under perpendicular fields, but stability remained largely unaffected. This can be attributed to the presence of strong flow forces along the scan direction, where both beams follow the same path, decoupling and minimizing the influence of effects of transverse EFs. In contrast, y‐BLP shows slight undulations under perpendicular EF, as the EF direction and offset axis are the same, leading to coupling. For xy‐BLP, both parallel and perpendicular EFs improved continuity, though stability was reduced under perpendicular EFs relative to the case of control and parallel EFs, for similar reasons as y‐BLP.

**FIGURE 7:**
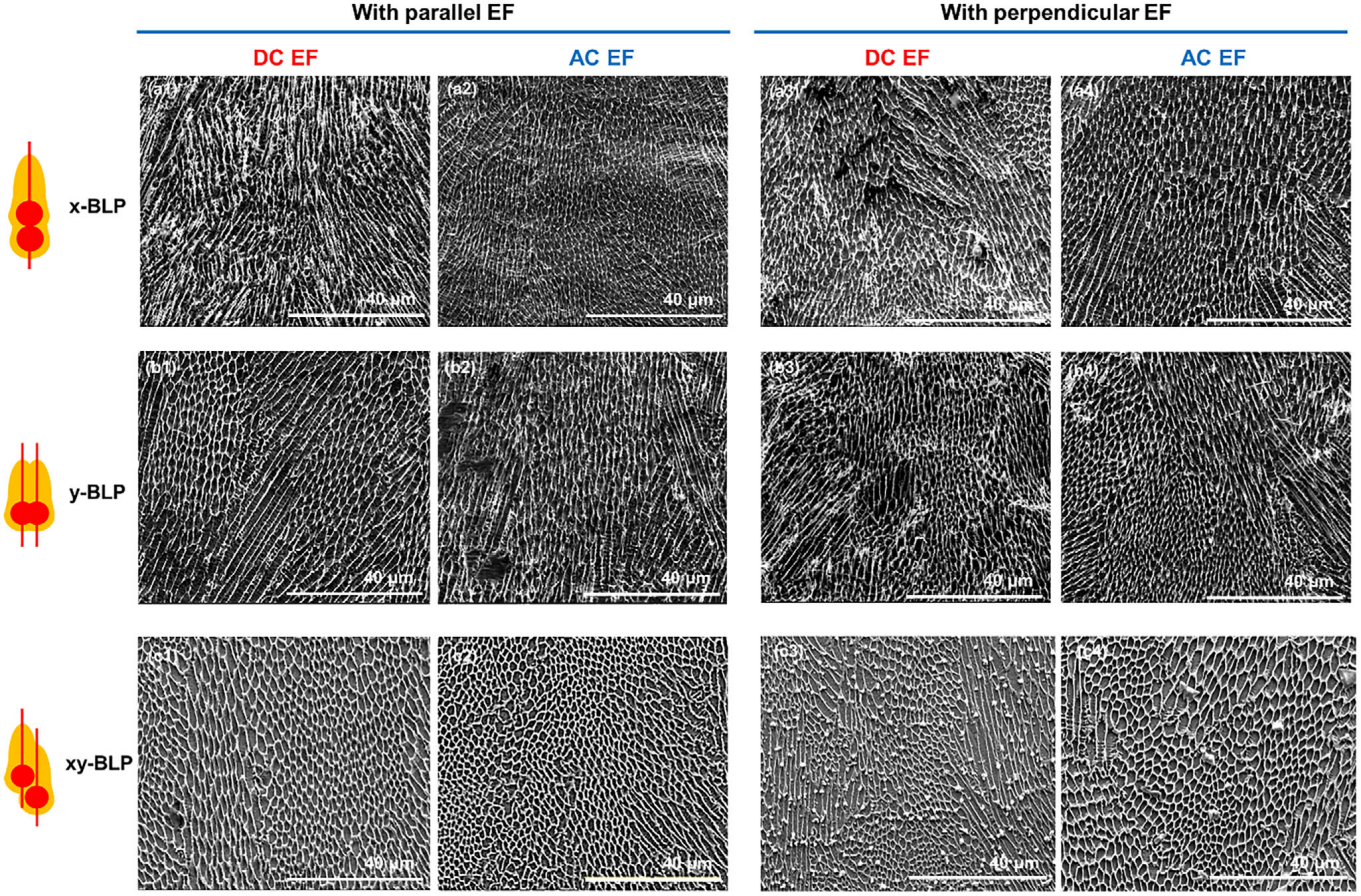
Cross‐sectional SEMs of resulting microstructures for (a) x‐BLP, (b) y‐BLP, and (c) xy‐BLP, under (1) parallel DC, (2) parallel AC, (3) perpendicular DC, and (4) perpendicular AC EFs.

Figure 7 shows SEM images of microstructures formed by different BLP configurations under the influence of parallel and perpendicular EFs (400 V DC and 400 V AC (at 6 kHz)). Upon utilization of parallel DC and AC EFs for x‐BLP (Figure 7a1,a2) and y‐BLP (Figure 7b1,b2) configurations, more equiaxed microstructures were observed compared to its control counterparts without an EF (Figure 5d). For perpendicular EFs, x‐BLP and y‐BLP microstructures comprises of a mix of columnar and equiaxed grains as seen in Figure 7a3,a4, and b3,b4. xy‐BLP configuration on the other hand, has the more equiaxed grains among the three BLP configurations without the application of an EF (Figure 5d–f). Upon introducing parallel EFs, xy‐BLP showed the highest distribution of equiaxed grains (Figure 7c1,c2), exceeding x‐BLP and y‐BLP. AC EFs consistently lead to a smaller and higher number of equiaxed grains compared to DC EFs (Figure 7c1,c2). Under perpendicular fields, xy‐BLP again showed significant equiaxed structures (Figure 7c3,c4). Moreover, consistently DC fields lead to a mixture of fine and coarse equiaxed grains (Figure 7c3), whereas AC fields generate more uniformly sized equiaxed grains (Figure 7c4).

### 2.3 Synergetic Structural Effects of Intensity Redistribution and EFs Across Length Scales

**FIGURE 8:**
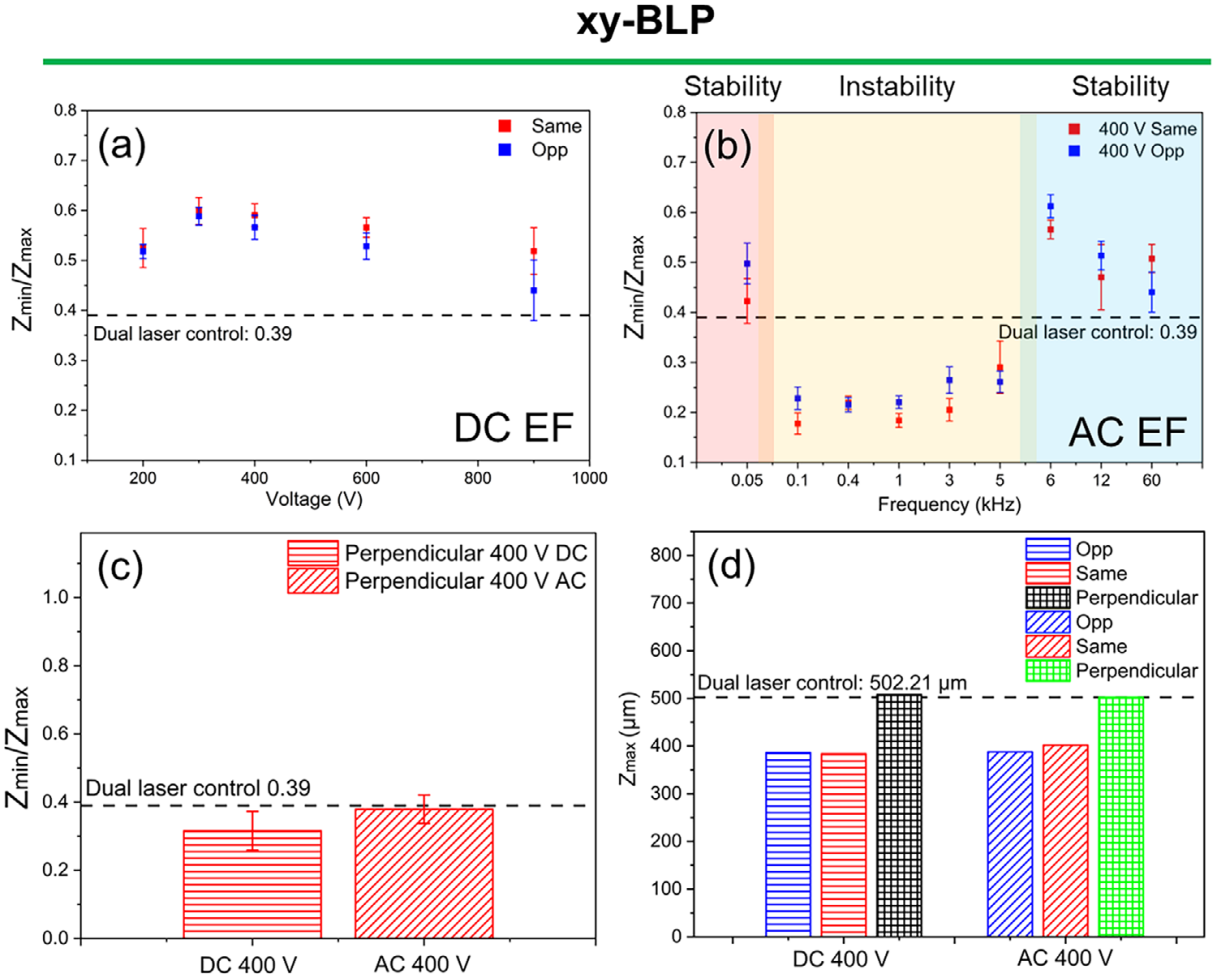
Assessment parameter $\left(\frac{Z_{min}}{Z_{max}}\right)$ for xy‐BLP with (a) parallel DC, (b) parallel AC, and (c) perpendicular DC and AC EFs. (d) Z_max_ values for EF‐assisted xy‐BLP.

Since xy‐BLP produced more refined microstructures than conventional ULP, and the addition of an EF both enhanced this refinement and visibly mitigated the discontinuities and instabilities typical of xy‐BLP, we next analyze EF‐assisted xy‐BLP structures in greater detail. First, macroscopic quantitative analyses are made on melt tracks to understand the effects of EF on laser processing. Figure 8a,b shows the assessment parameter $\left(\frac{Z_{min}}{Z_{max}}\right)$ for DC and AC EFs in xy‐BLP processes under the two parallel orientations for different applied potentials and frequencies. xy‐BLP with parallel DC EFs shows higher $\left(\frac{Z_{min}}{Z_{max}}\right)$ values than xy‐BLP without EFs, meaning that the melt track is continuous with no significant undulation. The $\left(\frac{Z_{min}}{Z_{max}}\right)$ assessment parameter increases up to ∼400 V and then decreases with increasing parallel DC voltage magnitudes (Figure 8a). Undulations emerge due to the break of the stable equilibria after a potential range of 300–400 V.

For AC EFs on xy‐BLP (Figure 8b), at extremely low frequencies (< 100 Hz) the $\left(\frac{Z_{min}}{Z_{max}}\right)$ is higher than xy‐BLP without EFs, indicating continuous melt tracks with improved stability (no major undulation present). Subsequently, at intermediate AC EF frequencies (100 Hz ≤ f ≤ 5 kHz), $\left(\frac{Z_{min}}{Z_{max}}\right)$ magnitudes are lower than its control counterpart (xy‐BLP without EF), indicating prominent instability in the form of distinct undulations in the melt track. At higher frequencies, i.e. > 5 kHz, $\left(\frac{Z_{min}}{Z_{max}}\right)$ is higher than the control, indicating a continuous melt track with no major undulations. Such behavior in stability transitions has been observed in the past for laser processing of SS316L upon frequency modulation [30]. Furthermore, melt oscillations in LPBF of SS316L have similar order of frequency [31], indicating that length scales of the meltpool can be governing factors for understanding the frequency transition behavior on the stability of the melt track.

Figure 8c shows $\left(\frac{Z_{min}}{Z_{max}}\right)$ for melt tracks formed with a perpendicular DC and AC EFs, indicating continuous yet unstable melt tracks. This continuous yet unstable behavior of melt tracks under perpendicular EFs is also indicated through additional assessment parameters reported in (Figure S6). Quantitatively, the perpendicular DC EF creates slightly more unstable (higher undulations) melt track compared to its control counterparts, as observed from Figure 8c. This is due to the tendency of a liquid front to move in the direction of the EF, which in the case of a perpendicular EF, is not in the direction of the scan/melt track [32]. However, since there exist strong flow forces in the scan direction, extensive movement of the melt perpendicular to the scan direction is negligible. Figure S6b,c reports higher standard deviation and coefficient of variation for DC EFs compared to that of its AC counterpart (closer to the control). In comparison, for parallel EFs at similar DC and AC parameters, stable melt tracks were observed with lower standard deviation and coefficient of variation (Figure S5c–f). Furthermore, the tendency to reduce the surface area of the melt results in the rise of Z_max_, for both DC and AC EFs, with DC slightly higher than the AC EFs and significantly higher than its parallel counterparts, as observed in Figure 8d. Therefore, perpendicular EFs (both DC and AC) do not help in reducing undulation, but support undulation. Additional assessment parameters are discussed in the Suppoting information, which indicates similar inferences as observed through the $\left(\frac{Z_{min}}{Z_{max}}\right)$ parameter. Investigations of surface topography of single scans are important, however, considering the complexity of the process, further works are required to see the upscaling potential and the effects of multiple scans and dimensions on the surface topography of bulids. Therefore, powerful modeling tools such as part scale‐modeling [33, 34] involving multiphysics can be incorporated to efficiently predict and understand such complex processes in higher dimensions.

**FIGURE 9:**
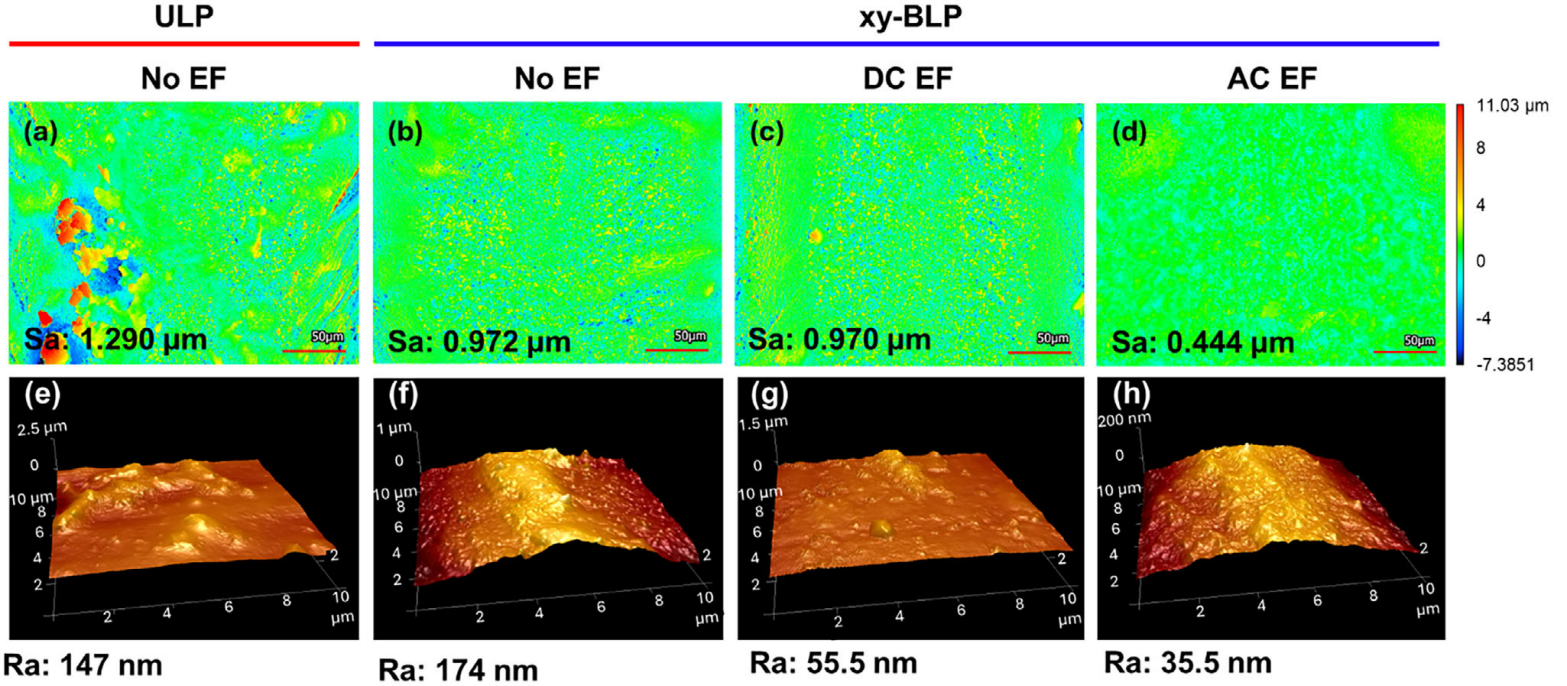
Microscale and nanoscale roughness determined through (a–d) confocal microscopy and (e,f) atomic force microscopy. (a,e) ULP without EF, (b,f) xy‐BLP without EF, (c,g) xy‐BLP with DC EF, (d,h) xy‐BLP with AC EF. The color scale corresponds to the normalized height profile for all confocal mappings.

To further investigate the effect of EFs on structure across different length scales, the micro‐ and nano‐scale surface roughness of the melt tracks was analyzed via confocal and atomic force microscopy (AFM) (Figure 9). Micro‐scale roughness shows the highest surface roughness (Sa) value of ∼1.29 µm for ULP samples without EFs, arising from the defects formed due to the high energy intensity of the single laser source (Figure 9a). xy‐BLP in the absence of an EF shows the second‐highest micro‐scale surface roughness of ∼0.972 µm (Figure 9b), owing to the observed macro‐structural discontinuity due to the coalescence phenomena (Figure 5e). xy‐BLP sample under a DC EF exhibited a slightly lower surface roughness of ∼0.970 µm (Figure 9c). On the other hand, the xy‐BLP sample under an AC EFs exhibited a noticeably smoother surface with a roughness of ∼0.444 µm (Figure 9d), consistent with the SEM observations (Figure 6c3,c4).

At the nano‐scale, AFM measurements indicate that the ULP samples without EFs show a surface roughness (R_a_) of ∼ 147 nm (Figure 9e). The xy‐BLP samples without EFs exhibit a higher R_a_ of ∼174 nm (Figure 9f), which is believed to arise from the highly complex coalescence phenomena. xy‐BLP samples with DC (Figure 9g) and AC (Figure 9h) EFs show R_a_ values of ∼55.5 nm and 35.5 nm, respectively. Therefore, choosing the right EF parameter, which in this case is AC EF at 400 V and 6 kHz, can produce better surface quality in terms of roughness on a macro, micro, and nanoscale compared to its control counterparts (ULP and xy‐BLP without EFs). Raman analysis of the melt tracks indicated the presence of typical components of SS316L, including α‐FeOOH [35], γ‐Fe_2_O [36, 37], Fe_2_O, and γ‐FeOOH [35], FeO, and Fe_3_O_4_, across all samples (Figure S15). Moreover, noticeable differences in XPS spectra could not be determined between the melt tracks prepared without and with an EF (Figure S16), indicating that the chemical composition was largely not affected by the application of an external EFs [38, 39].

**FIGURE 10:**
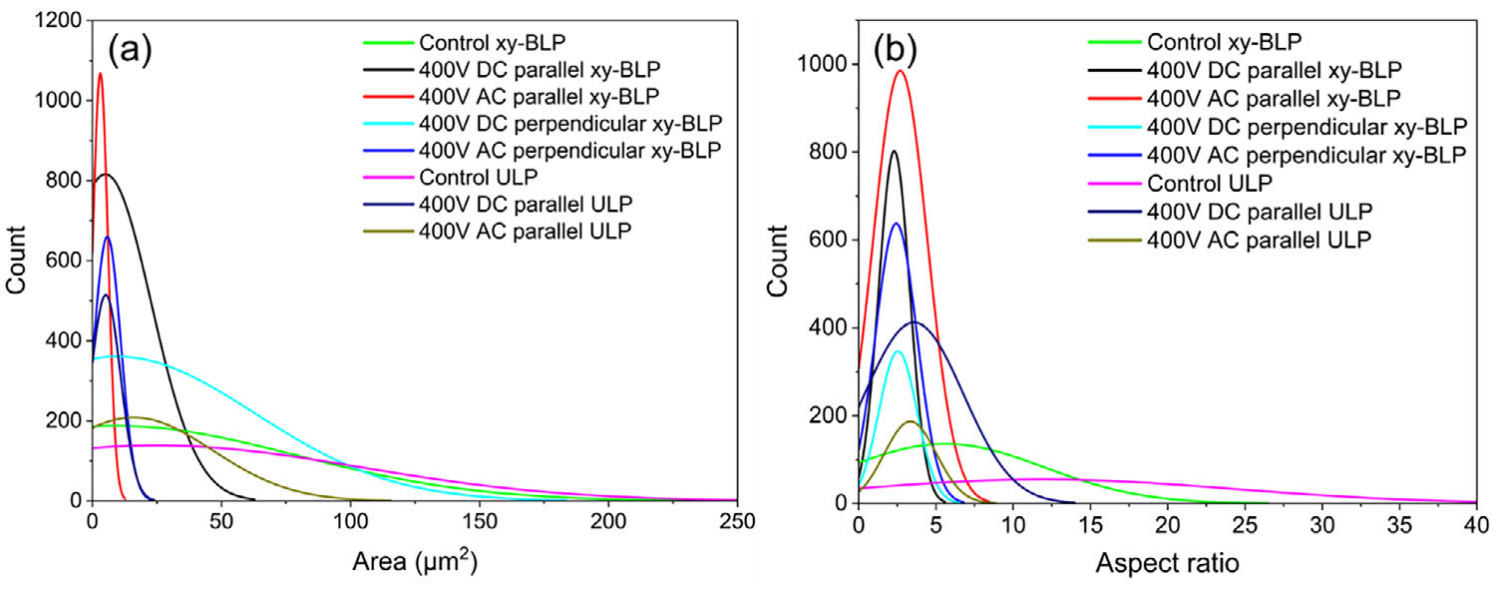
Distribution of microstructural grain (a) area and (b) aspect ratio of melt tracks.

Beyond surface‐level improvements, EF effects within the bulk were assessed through quantitative microstructural analysis. Grain size distributions at the cross‐sectional track center were obtained by ImageJ processing (Figures S6–S8 and Table S1), with results summarized in Figure 10a. Without an EF, both control ULP (magenta) and xy‐BLP (light green) exhibited large average grain areas of 24.806 and 8.970 µm^2^ with high deviation of 79.373 and 70.309 µm^2^, respectively. This confirms that even without EFs, advanced beam‐shaping techniques such as BLP lead to slightly less coarsened grains. Under a parallel EF, the average grain size further reduced in both ULP (DC: navy, AC: green) and BLP (DC: black, AC: red). The combination of BLP with parallel EFs led to an average grain size of ∼5 µm^2^ with a standard deviation of 17.7 µm^2^ for DC, while AC further reduced it to ∼3 µm^2^ with 2.9 µm^2^ deviation. In contrast, perpendicular EFs in xy‐BLP (DC: light blue, AC: blue) also reduced grain size to 5.7 and 9.5 µm^2^ with deviations of 53.4 and 5.198 µm^2^, respectively, but less effectively than parallel EFs.

To further quantify the microstructure, the ratio of the longer length to the shorter width of the grain, or the aspect ratios (AR), was calculated (Figure 10b). Columnar grains will show a higher AR number, whereas equiaxed grains shall demonstrate lower numbers. Without the application of an EF, control ULP samples exhibited highly columnar grains with the highest mean AR of 11.919 with a standard deviation of 12.348. xy‐BLP lead to the slight formation of equiaxed grains, with a mean AR of 5.609 and a standard deviation of 6.416. The introduction of parallel DC and AC EFs in ULP, the AR reduces to 3.578 and 3.343 with standard deviations of 3.193 and 1.690, respectively, indicating strong promotion of equiaxed growth. Upon the combination of xy‐BLP with parallel DC and AC EFs, a further refinement is indicated with AR values of 2.297 and 2.689 with standard deviations of 1.007 and 1.763, respectively. While perpendicular DC and AC EFs with xy‐BLP leads to significantly reduced AR values of 2.542 and 2.427 with standard deviations of 1.237 and 1.338, respectively, compared to ULP, the lower count values compared to parallel EF xy‐BLP indicate larger grains. This quantitatively shows that parallel EFs particularly drive significant grain refinement and facilitate equiaxed grain formations, with strong synergistic effects when combined with BLP. Therefore, utilizing combined EF and BLP methods leading to smaller equiaxed grain structures may be applied toward strengthening mechanisms for enhanced mechanical properties [22, 40, 41].

**FIGURE 11:**
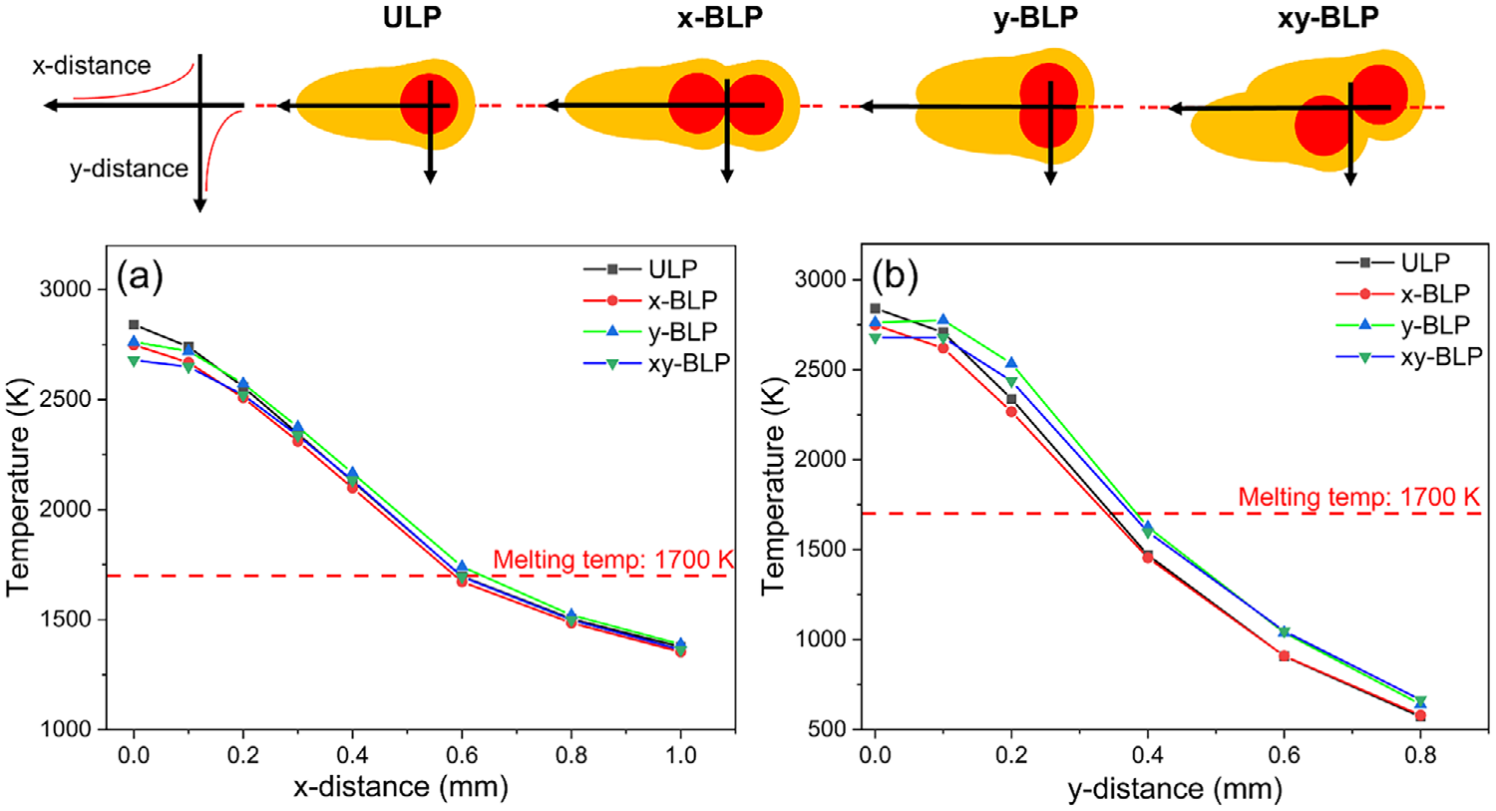
FEM estimated temperature gradients for ULP and various BLP configurations along (a) x‐ and (b) y‐distances. Top images simply show schematic illustrations of the melt pool under different processing configurations.

Equiaxed grain formation during BLP is typically attributed to changes in temperature gradients compared to ULP. Microstructural grain size and morphology are dependent on the temperature gradient at the solid–liquid interface (G in K mm^−1^) and the solidification front growth rate (R in mm s^−1^). Higher cooling rates or G×R (K s^−1^) yield finer grains, while lower G/R (K s mm^−2^) enables more equiaxed grains. Therefore, reducing the temperature difference across the solid–liquid interface lowers G/R and favors equiaxed growth. Compared to ULP, xy‐BLP forms more equiaxed grains, and the addition of an EF further refines the microstructure. FEM estimations of the temperature gradients show that ULP exhibits a slightly higher temperature gradient than BLP (Figure 11). However, this difference is small, suggesting that equiaxed grains observed in BLP arise from a combined effect of reduced gradients and altered melt pool geometry [25]. Microstructural analyses further revealed that EF‐assisted BLP induces significant grain refinement, suggesting mechanisms beyond thermal and geometrical control alone. Electrical stimuli can influence the solid–liquid interface, leading to further variations in temperature gradients [42], melt pool shape or flows [25], surface energies, and various static or alternating magnetic fields [11] that may collectively contribute. While these observations confirm that EFs strongly influence grain formation and growth, the underlying mechanisms and their interplay remain unresolved and require further investigation.

## 3 Conclusion

This study demonstrates the effects of external EFs across multiple length scales during laser scanning of powder metals. Several key conclusions can be drawn:

- Application of EFs improves continuity and stability of resolidified tracks, even under complex xy‐BLP beam shapes, which are susceptible to severe discontinuities.
- Parallel EFs stabilize melt tracks, while perpendicular EFs can destabilize them depending on the relationship between field direction, beam geometry, and scan path.
- DC EFs consistently stabilize melt tracks, whereas AC EFs provide stability only at very high (>5 kHz) or very low (<100 Hz) frequencies.
- AC EFs combined with xy‐BLP reduce roughness across length scales, including nanometer features, enabling higher‐quality surfaces.
- BLP alone improves microstructure over ULP, but EFs promote stronger refinement toward equiaxed grains, with AC EFs outperforming DC EFs.
- Random particle geometries and oxide layers cause non‐uniform field distributions, contributing to complex physical phenomena.

The implications of external‐EFs beyond 0D laser processing were successfully demonstrated. These findings collectively provide one of the first systematic demonstrations of EF‐assisted beam shaping in powder‐metal laser processing. Moreover, a number of laser processing parameters, such as laser power, scan speed, and powder bed thickness, which can affect the process when investigated with EFs requires extensive research. However, by demonstrating feasibility and establishing how field parameters, such as type, orientation, and frequency, govern stability, surface finish, and grain refinement, this work benchmarks the potential of EFs as a new axis for adaptive control. Most results indicate surface tension to be the major contributing mechanism, however, additional studies are required. Therefore, future efforts should focus on unraveling the underlying physics of field–melt interactions and extending this framework toward multi‐field or real‐time adaptive control in powder bed fusion and other advanced laser systems.

## 4 Experimental Section

A ∼400 µm layer of stainless steel (SS316L) powder (average diameter ∼40 µm, Alfa Aesar, U.S.A) was deposited on a SS316L substrate (∼1.22 mm thick, McMaster‐Carr, U.S.A), both as received. The substrate which further positioned on a polylactic acid base to electrically insulate from the surroundings. EFs, both DC and AC, were applied across the powder bed using a pair of copper electrodes (bottom left of Figure 4a), such that they did not contact the conductive SS316L substrate to realize a non‐contact configuration. All experiments were performed with a fixed electrode distance of ∼20 mm, which enables calculation of the electric field strength as: $\mathrm{Electric}\;\mathrm{field}\;\mathrm{strength}\;\;\left(\frac{V}{m}\right)=\frac{\mathrm{Applied}\;\mathrm{voltage}\;(V)}{\mathrm{distance}\;\mathrm{between}\;\mathrm{the}\;\mathrm{electrides}(m)}=\frac{\mathrm{Applied}\;\mathrm{voltage}\;(V)}{20\times 10^{-3}\left(m\right)}=\;\mathrm{Applied}\;\mathrm{voltage}\times 50\left(\frac{V}{m}\right)$. The setup was ensured for no current flow through the SS316L substrate by using a multimeter (upto nA).

For laser experiments, two ∼1064‐nm continuous‐wave Nd:YAG lasers (IPG photonics, U.S.A), each with a power of 100 W, were utilized. All experiments were conducted in ambient air. Experiments conducted with a single laser were termed unimodal laser processing (ULP), whereas those with two laser systems were termed bimodal laser processing (BLP). Each laser beam possessed a Gaussian distribution and was independently focused onto the same powder bed to a spot size of ∼220 µm using separate galvanometer scanners (Raylase GmbH, Germany). By superimposing the two beams with controlled spatial offsets, arbitrary beam profiles could be constructed (Figure 4b). According to the methodology reported by Zhang et al. [29], BLP experiments were conducted with x‐ and y‐offsets. x‐BLP configuration has one beam following another at an x‐offset of ∼220 µm. y‐BLP configuration has two beams side by side at a y‐offset of ∼110 µm. xy‐BLP configuration has two beams at an x‐ and y‐offset of ∼220 and ∼110 µm, respectively. The mentioned BLP configurations have been explored to have a systematic understanding of which bimodal distribution can control high thermal gradients and offer superior microstructural properties [24]. Laser parameters, such as power and scan speed, as well as the spatial and temporal offsets between beams, were digitally controlled via a computer (Figure 4a). Laser parameters were set at a scan speed of 40 mm/s and a total laser irradiation power of 200 W (100 W for each laser) for all tracks shown in the text, unless otherwise mentioned. Rationale for this parameter is outlined in Section 1 of (Figures S2 and S3).

The EFs were applied to the powder bed during laser processing using a high‐voltage DC operational amplifier (OP‐AMP, PZ‐70, Burleigh, U.S.A), driven by either a DC power supply (PS‐303D, Dazheng, China) or a function generator (3311A, Hewlett Packard, U.S.A). Relative to the laser scan direction shown in Figure 4b, different EF orientations were applied (Figure 4c). Parallel EFs refer to fields aligned with (same) or opposite (opp) to the scan, whereas perpendicular EFs are oriented across the scan direction. Voltage was monitored through an oscilloscope (DPO 2022B, Tektronix, U.S.A). DC EFs were controlled by changing the root mean square DC voltage (V_rms_), as shown in Figure 4d. For AC EFs, the frequency was set by the function generator, while the amplitude (V_Ampl_) was set to be constant at ∼100 ± 5 V. The V_rms_ was controlled by tuning the offset voltage (V_offset_). V_rms_ (deviation: ± 5 V) for both DC and AC EFs are reported.

For qualitative structural characterization, a SEM (Quanta, Thermo Fisher, U.S.A) was utilized to observe the solidified melt tracks and their microstructure. For microstructural observations, the tracks were cross‐sectioned using a diamond cutter with cooling fluid and mounted by hot pressing (SimpliMet 4000 mounting system, Buehler, Switzerland). The samples were surface‐polished sequentially with emery paper up to 4000 grit to a mirror‐like finish, then etched for ∼20 s. The etchant consisted of 15 mL HCl (12 m), 10 mL CH_3_COOH (17.4 m), 10 mL HNO_3_ (15.7 m), and a drop of glycerol (≥ 99.5% purity). After etching, samples were rinsed with distilled water and dried prior to observation. All observations were conducted at a fixed location vertically above the melt track center and approximately halfway between the center and the top surface (Figure S7). The microstructures were quantified using standard ImageJ software procedures, which followed the ASTM E1382 standard [43].

**FIGURE 12:**
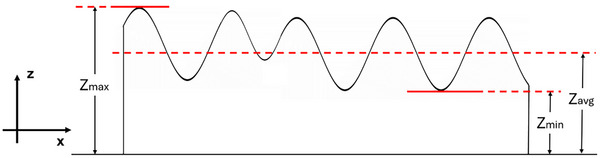
Illustration of an obtained undulated melt track height profile with assessment variables defined (not to scale).

For quantitative structural characterization, a laser confocal microscope (Keyence VK‐X3050, Japan) was utilized to obtain height profiles of the resolidified structure. The maximum, minimum, and average heights, denoted as Z_max_, Z_min,_ and Z_avg_, respectively, were extracted to assess the surface profiles (Figure 12). A number of assessment parameters can be defined to account for the surface profile (Equations (1) and (2)). A smaller $\left(\frac{Z_{min}}{Z_{max}}\right)$ value closer to 0 indicates that the troughs of the undulations in the melt track are away from the crests, resulting in a strong wavy undulated track. Each experiment was repeated 10 times with the same process parameters, and the mean is reported with a standard error bar. Note that the start and end of the resolidified structures were excluded from analyses, leaving an approximate length of ∼8 mm over which the assessment parameters are analyzed. Furthermore, since the first undulation is a characteristic of all experiments, it is omitted from the data set. Additional assessment parameters of standard deviation and coefficient of variation (defined as population standard deviation/population mean) are also utilized.

(1) $$\frac{Z_{min}}{Z_{max}}\;\left\{\begin{array}{c} closer\;to\;1;less\;waviness\\ away\;from\;1;more\;waviness \end{array}\right\}$$

(2) $$\frac{Z_{avg}}{Z_{max}}\left\{\begin{array}{c} closer\;to\;1;less\;waviness\\ away\;from\;1;more\;waviness \end{array}\right\}$$

## Funding

This work was jointly funded by Princeton University and the Office of Naval Research, under Sponsor Award Number K004522‐00‐S01 (PRIME ONR # N68335‐24‐C‐0039). The characterizations were conducted with the support of the Princeton Imaging and Analysis Center (IAC) which is partially supported by the Princeton Center for Complex Materials, a National Science Foundation (NSF)‐MRSEC program (DMR‐2011750).

## Conflicts of Interest

The authors declare no conflict of interest.

## Supporting information




**Supporting File**: advs74247‐sup‐0001‐SuppMat.docx.

## Data Availability

The data that support the findings of this study are available from the corresponding author upon reasonable request.

## References

[1] I. M. Kusoglu , B. Gökce , and S. Barcikowski , “Research Trends in Laser Powder Bed Fusion of Al Alloys Within The Last Decade,” Additive Manufacturing 36 (2020): 101489, 10.1016/j.addma.2020.101489.

[2] I. Yadroitsev , I. Yadroitsava , A. Du Plessis , and E. MacDonald , Fundamentals of Laser Powder Bed Fusion of Metals. (Elsevier, 2021).

[3] W. E. King , A. T. Anderson , R. M. Ferencz , et al., “Laser Powder Bed Fusion Additive Manufacturing of Metals; Physics, Computational, and Materials Challenges,” Applied Physics Reviews 2 (2015): 041304, 10.1063/1.4937809.

[4] N. D. Dejene and H. G. Lemu , “Current Status And Challenges of Powder Bed Fusion‐Based Metal Additive Manufacturing: Literature Review,” Metals 13 (2023): 424, 10.3390/met13020424.

[5] C. Wei and L. Li , “Recent Progress And Scientific Challenges In Multi‐Material Additive Manufacturing Via Laser‐Based Powder Bed Fusion,” Virtual and Physical Prototyping 16 (2021): 347–371, 10.1080/17452759.2021.1928520.

[6] S. R. Narasimharaju , W. Zeng , T. L. See , et al., “A Comprehensive Review on Laser Powder Bed Fusion Of Steels: Processing, Microstructure, Defects And Control Methods, Mechanical Properties, Current Challenges and Future Trends,” Journal of Manufacturing Processes 75 (2022): 375–414, 10.1016/j.jmapro.2021.12.033.

[7] S. Rott , A. Ladewig , K. Friedberger , J. Casper , M. Full , and J. H. Schleifenbaum , “Surface Roughness in Laser Powder Bed Fusion—Interdependency of Surface Orientation and Laser Incidence,” Additive Manufacturing 36 (2020): 101437, 10.1016/j.addma.2020.101437.

[8] Z. Qu , Z. J. Zhang , Y. K. Zhu , et al., “Coupling Effects of Microstructure and Defects on the Fatigue Properties of Laser Powder Bed Fusion Ti‐6Al‐4 V,” Additive Manufacturing 61 (2023): 103355, 10.1016/j.addma.2022.103355.

[9] C. Du , Y. Zhao , J. Jiang , et al., “Pore Defects in Laser Powder Bed Fusion: Formation Mechanism, Control Method, and Perspectives,” Journal of Alloys and Compounds 944 (2023): 169215, 10.1016/j.jallcom.2023.169215.

[10] I. Yadroitsev , I. Yadroitsava , and A. Du Plessis , Fundamentals of Laser Powder Bed Fusion of Metals. (Elsevier, 2021): 15–38.

[11] H. Zhou , C. Song , Y. Yang , et al., “The Microstructure and Properties Evolution of SS316L Fabricated by Magnetic Field‐Assisted Laser Powder Bed Fusion,” Materials Science and Engineering: A 845 (2022): 143216, 10.1016/j.msea.2022.143216.

[12] A. Guo , R. Tang , S. Guo , et al., “Acoustic Field‐Assisted Powder Bed Fusion of Tungsten Carbide‐Reinforced 316L Stainless Steel Composites,” Journal of Materials Research and Technology 26 (2023): 5488–5502, 10.1016/j.jmrt.2023.08.271.

[13] H. Conrad , “Influence of an Electric or Magnetic Field on the Liquid–Solid Transformation in Materials and on the Microstructure of the Solid,” Materials Science and Engineering: A 287 (2000): 205–212, 10.1016/S0921-5093(00)00777-2.

[14] R. Qin and B. Zhou , “Effect of Electric Current Pulses on Grain Size in Castings,” International Journal of Non‐Equilibrium Processing(UK) 11 (1998): 77–86.

[15] R. Digilov , “Charge‐Induced Modification of Contact Angle: The Secondary Electrocapillary Effect,” Langmuir 16 (2000): 6719–6723, 10.1021/la991308a.

[16] X. Yang , Z. He , J. Yu , Y. Zhang , L. Yuan , and F. Mao , “Influence of Interface Electric Field on Interaction Between Molten Iron and Refractory Interface,” Ceramics International 46 (2020): 10180–10185, 10.1016/j.ceramint.2020.01.009.

[17] G. Holló , N. J. Suematsu , E. Ginder , and I. Lagzi , “Electric Field Assisted Motion of a Mercury Droplet,” Scientific reports 11 (2021): 2753. 33531526 10.1038/s41598-020-80375-1 PMC7854757

[18] Y. Du and C. B. Arnold , “Prediction of the Inter‐Track Bonding During the Dual‐Laser Powder Bed Fusion,” Journal of Manufacturing Processes 120 (2024): 911–919, 10.1016/j.jmapro.2024.04.087.

[19] M. C. Sow , T. De Terris , O. Castelnau , et al., “Influence of Beam Diameter on Laser Powder Bed Fusion (L‐PBF) Process,” Additive Manufacturing 36 (2020): 101532, 10.1016/j.addma.2020.101532.

[20] U. S. Bertoli , B. E. MacDonald , and J. M. Schoenung , “Stability of Cellular Microstructure in Laser Powder Bed Fusion of 316L Stainless Steel,” Materials Science and Engineering: A 739 (2019): 109–117, 10.1016/j.msea.2018.10.051.

[21] N. Coniglio and C. Cross , “Initiation and Growth Mechanisms for Weld Solidification Cracking,” International Materials Reviews 58 (2013): 375–397, 10.1179/1743280413Y.0000000020.

[22] Z. Hu , S. Gao , L. Zhang , et al., “Micro Laser Powder Bed Fusion of Stainless Steel 316L: Cellular Structure, Grain Characteristics, and Mechanical Properties,” Materials Science and Engineering: A 848 (2022): 143345, 10.1016/j.msea.2022.143345.

[23] Y. M. Wang , T. Voisin , J. T. McKeown , et al., “Additively Manufactured Hierarchical Stainless Steels With High Strength and Ductility,” Nature Materials 17 (2018): 63–71. 29115290 10.1038/nmat5021

[24] Z. Liu , Y. Yang , Y. Xiao , et al., “Investigation of 316L Microstructure Evolution Mechanism and Mechanical Properties in Dual‐Laser Powder Bed Fusion With Controllable Remelting Time Interval,” Materials & Design 239 (2024): 112761, 10.1016/j.matdes.2024.112761.

[25] T. T. Roehling , S. S. Q. Wu , S. A. Khairallah , et al., “Modulating Laser Intensity Profile Ellipticity for Microstructural Control During Metal Additive Manufacturing,” Acta Materialia 128 (2017): 197–206, 10.1016/j.actamat.2017.02.025.

[26] J. Karimi , C. Zhao , and K. Prashanth , “Massive Transformation in Dual‐Laser Powder Bed Fusion of Ti_6_Al_4_V Alloys,” Journal of Manufacturing Processes 119 (2024): 282–292, 10.1016/j.jmapro.2024.03.083.

[27] Z. Liu , C. Song , X. Han , Y. Xiao , K. Liu , and Y. Yang , “Effect of Dual‐Laser Powder Bed Fusion on Surface Quality, Internal Defects and Properties of 316L Stainless Steel,” Journal of Materials Research and Technology 27 (2023): 5681–5691, 10.1016/j.jmrt.2023.10.221.

[28] W. Zhang , W. Hou , L. Deike , and C. Arnold , “Understanding the Rayleigh Instability in Humping Phenomenon During Laser Powder Bed Fusion Process,” International Journal of Extreme Manufacturing 4 (2022): 015201, 10.1088/2631-7990/ac466d.

[29] W. Zhang , W. Hou , L. Deike , and C. B. Arnold , “Using a Dual‐Laser System to Create Periodic Coalescence in Laser Powder Bed Fusion,” Acta Materialia 201 (2020): 14–22, 10.1016/j.actamat.2020.09.071.

[30] M. Rupp , K. Schwarzkopf , M. Döring , S. Hayashi , M. Schmidt , and C. B. Arnold , “Laser Driven Melt Pool Resonances Through Dynamically Oscillating Energy Inputs,” Journal of Manufacturing Processes 131 (2024): 1624–1630, 10.1016/j.jmapro.2024.09.100.

[31] L. Caprio , A. G. Demir , and B. Previtali , “Observing Molten Pool Surface Oscillations During Keyhole Processing in Laser Powder Bed Fusion as a Novel Method to Estimate the Penetration Depth,” Additive Manufacturing 36 (2020): 101470, 10.1016/j.addma.2020.101470.

[32] A. R. Thiam , N. Bremond , and J. Bibette , “Breaking of an Emulsion Under an Ac Electric Field,” Physical Review Letters 102 (2009): 188304, 10.1103/PhysRevLett.102.188304. 19518918

[33] W. Lei , H. Jiabao , S. Shuoqing , et al., “Part‐Scale Multi‐Physics Model for Laser Powder Bed Fusion Process,” Cell Reports Physical Science 6 (2025): 102674, 10.1016/j.xcrp.2025.102674.

[34] M. Zheng , L. Wei , J. Chen , et al., “A Novel Method for the Molten Pool and Porosity Formation Modelling in Selective Laser Melting,” International Journal of Heat and Mass Transfer 140 (2019): 1091–1105, 10.1016/j.ijheatmasstransfer.2019.06.038.

[35] R. J. Thibeau , C. W. Brown , and R. H. Heidersbach , “Raman Spectra of Possible Corrosion Products of Iron,” Applied spectroscopy 32 (1978): 532–535, 10.1366/000370278774330739.

[36] I. Chourpa , L. Douziech‐Eyrolles , L. Ngaboni‐Okassa , et al., “Molecular Composition of Iron Oxide Nanoparticles, Precursors for Magnetic Drug Targeting, as Characterized by Confocal Raman Microspectroscopy,” The Analyst 130 (2005): 1395–1403, 10.1039/B419004A. 16172665

[37] M. H. Sousa , F. A. Tourinho , and J. C. Rubim , “Use of Raman Micro‐Spectroscopy in the Characterization of M^II^Fe_2_O_4_ (M = Fe, Zn) Electric Double Layer Ferrofluids,” Journal of Raman Spectroscopy 31 (2000): 185–191, 10.1002/(SICI)1097-4555(200003)31:3<185::AID-JRS511>3.0.CO;2-B.

[38] W. T. Choi , K. Oh , P. M. Singh , V. Breedveld , and D. W. Hess , “Hydrophobicity and Improved Localized Corrosion Resistance of Grain Boundary Etched Stainless Steel in Chloride‐Containing Environment,” Journal of The Electrochemical Society 164 (2017): C61–C65, 10.1149/2.1271702jes.

[39] W. T. Choi , K. Oh , P. M. Singh , V. Breedveld , and D. W. Hess , “Wettability Control of Stainless Steel Surfaces via Evolution of Intrinsic Grain Structures,” Journal of Materials Science 51 (2016): 5196–5206, 10.1007/s10853-016-9821-y.

[40] H. Sohrabpoor , V. Salarvand , R. Lupoi , et al., “Microstructural and Mechanical Evaluation of Post‐Processed SS 316L Manufactured by Laser‐Based Powder Bed Fusion,” Journal of Materials Research and Technology 12 (2021): 210–220, 10.1016/j.jmrt.2021.02.090.

[41] H. C. Hyer and C. M. Petrie , “Effect of Powder Layer Thickness on the Microstructural Development of Additively Manufactured SS316,” Journal of Manufacturing Processes 76 (2022): 666–674, 10.1016/j.jmapro.2022.02.047.

[42] K. Deshmukh , A. Riensche , B. Bevans , et al., “Effect of Processing Parameters and Thermal History on Microstructure Evolution and Functional Properties in Laser Powder Bed Fusion of 316L,” Materials & Design 244 (2024): 113136, 10.1016/j.matdes.2024.113136.

[43] C. de Souza Pereira , E. E. de Mello Oliveira , and T. G. de Paula , Microstructural Characterization of TiO_2_ Ceramics Substrates Using Image Analysis Software ImageJ. (Instituto de Engenharia Nuclear: Progress Report, 2021).

