# Supplementary material for: Multi‐Scale Structural Effects of External Electrical Fields of Melt Tracks in Laser Powder Bed Fusion
Source: Adv Sci (Weinh). 2026 Feb 8;13(21):e18344. doi: 10.1002/advs.202518344 (PMC13073313; doi:10.1002/advs.202518344)
Supplement: Supplementary file 1 — Supporting File: advs74247‐sup‐0001‐SuppMat.docx. [file ADVS-13-e18344-s001.docx]

Supporting Information

Multi-scale structural effects of external electrical fields of melt tracks in laser powder bed fusion

Ankit Das, Shuichiro Hayashi, Craig B. Arnold*

General experimental and characterization parameters


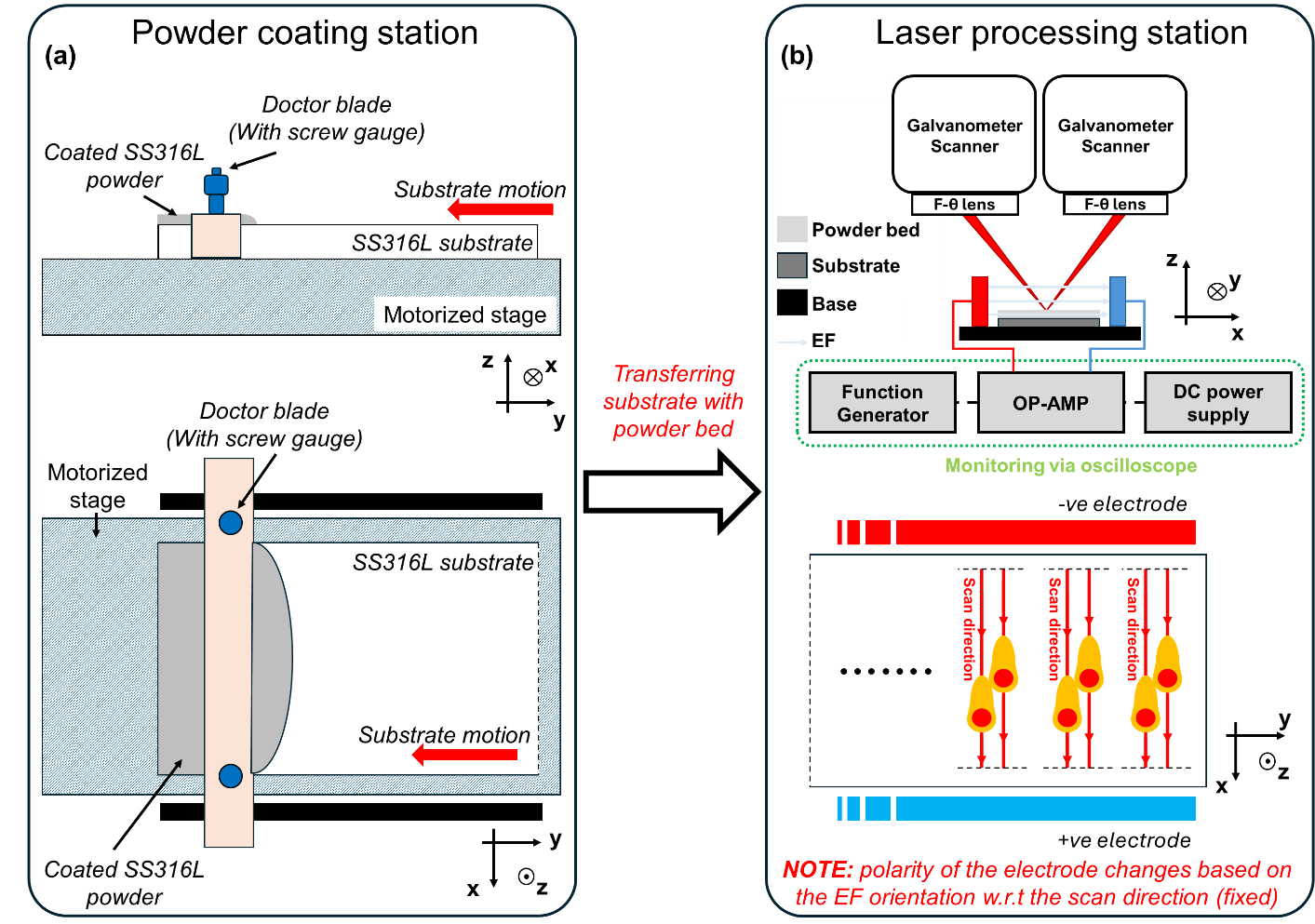


Figure S1: Detailed schematic of the experimental setups used for conducting the experiments demonstrated in this work. (a) the powder coating station, and (b) the laser processing station equipped with lasers and EFs.

SEM parameters for imaging melt lines utilized an SE detector, with an accelerating voltage: 10 kV, magnification: 60×, WD: 9.1-9.4 mm (depending on the sample) and a spot size of 5. SEM parameters for imaging microstructures (grains) utilized an SE detector, with an accelerating voltage: 15 kV, magnification: 1500×, WD: 8.9-10.4 mm (depending on the sample) and a spot size of 5. For AFM experimentations, tapping mode was utilized. A scan area of 10×10 μm^2^ was investigated. The scan rate and pixel counts were 0.5 Hz and 256×256 for the AFM scans.

1. **Preliminary studies on process parameters**


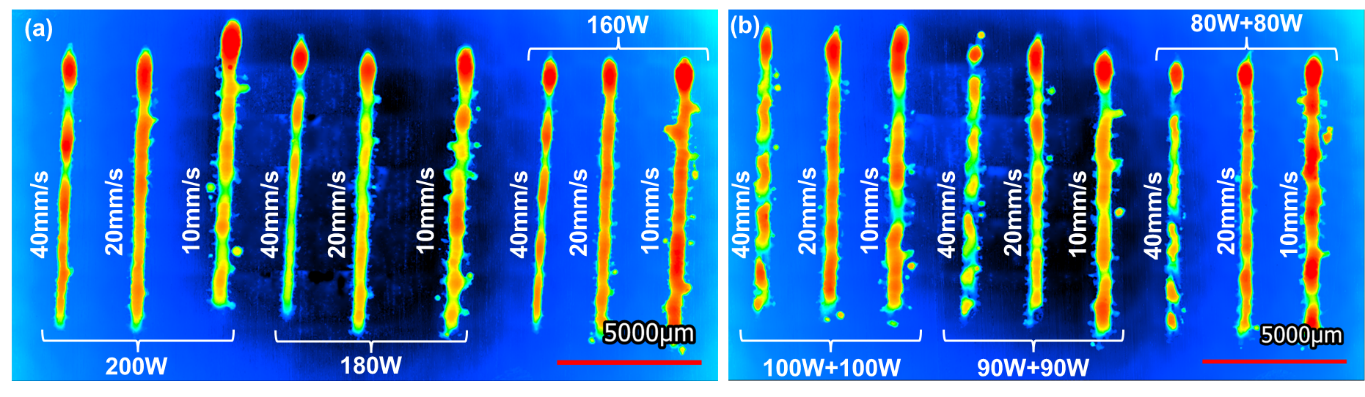


**Figure** S2: Confocal images of solidified melt tracks of SS 316L at varying scan speeds without EF; (a) Single laser irradiation, and (b) dual laser irradiation.

ULP and BLP process parameters affect the melt track performance in terms of its stability and continuity. Confocal and SEM images of melt tracks under different scan speeds without EFs are observed to determine laser parameters (**Figures** S2 and S3). Decrease in laser power has shown increase in porosity ^1^, affecting the melt track stability. Experiments conducted with x-BLP shows stable melt tracks at low speeds of 10 and 20 mm/s whereas at 40 mm/s the melt track shows slight undulations due to a Rayleigh instability (**Figure** S3(b1-b3)). y-BLP experiments demonstrate more stable melt track compared to its x-counterpart, but with a wider melt track due to the side by side orientation of the meltpools. Finally, the xy-BLP melt tracks show distinct phenomena at various speeds. At low speeds of 10 and 20 mm/s, stable melt tracks are realized, whereas at 40 mm/s, discontinuities are observed due to periodic coalescence of meltpools (**Figure** S3(d)). Increase in laser power also leads to continuous melt tracks as observed in **Figure** S3. Therefore, it can be argued that simply increasing laser power or reducing the scan speed might be a solution to the xy-BLP macrostructural instabilities and resulting undulations in the melt track (seen from **Figure** S3(d1,d2). However, increase in laser power or decrease in scan speed, increases the denudation width ^2^. Leading to process limitations. Therefore, higher scan speeds are desired, but utilizing higher speeds at the same laser power increases the melt track undulations and discontinuities in xy-BLP melt track compared to that of ULP, thereby limiting the process speed. Consequently, considering the problems associated with denudation width and heat affected zone in 10 mm/s and 20 mm/s processes, this research further utilizes a scan speed of 40 mm/s under a total laser irradiation power of 200 W and serves as the control for further investigations. It is important to note that these paramters are not similar to those as observed in typical LPBF experiments conducted in the literature, where a laser power of 200 W is coupled with scan speeds ≥ 300 mm/s on a powder bed layer of ≥ 20 μm thickness ^3,4^. However, the BLP experiments are different in nature, two beams with coalescing meltpools and are similar to the previous works of Du and Arnold^4^, and Zhang et al. ^3^. Likewise, the parameters have been optimized as presented earlier.

The melt track widths decrease with decrease in laser power and increase in scan speed. Since increasing scan speed and decreasing the laser power reduces the energy input, the effective melt pool is smaller, thus narrower melt tracks (**Figure** S2). The melt track widths of the ULP and BLP are visually comparable at similar laser power and scan speed as seen from **Figure** S3. However, upon measurement, the y and xy-BLP melt tracks are a little wider (by ~ 100 μm) than the ULP melt tracks. This observation is in line with the y and xy-BLP meltpool configuration, where the two lasers have a y-offset of ~110 μm, leading to a melt pool overlap of ~110 μm, and resulting in a wider melt track than that of ULP. **Figure** S3(d3) also shows defects such as discontinuity in melt tracks and balling.

**
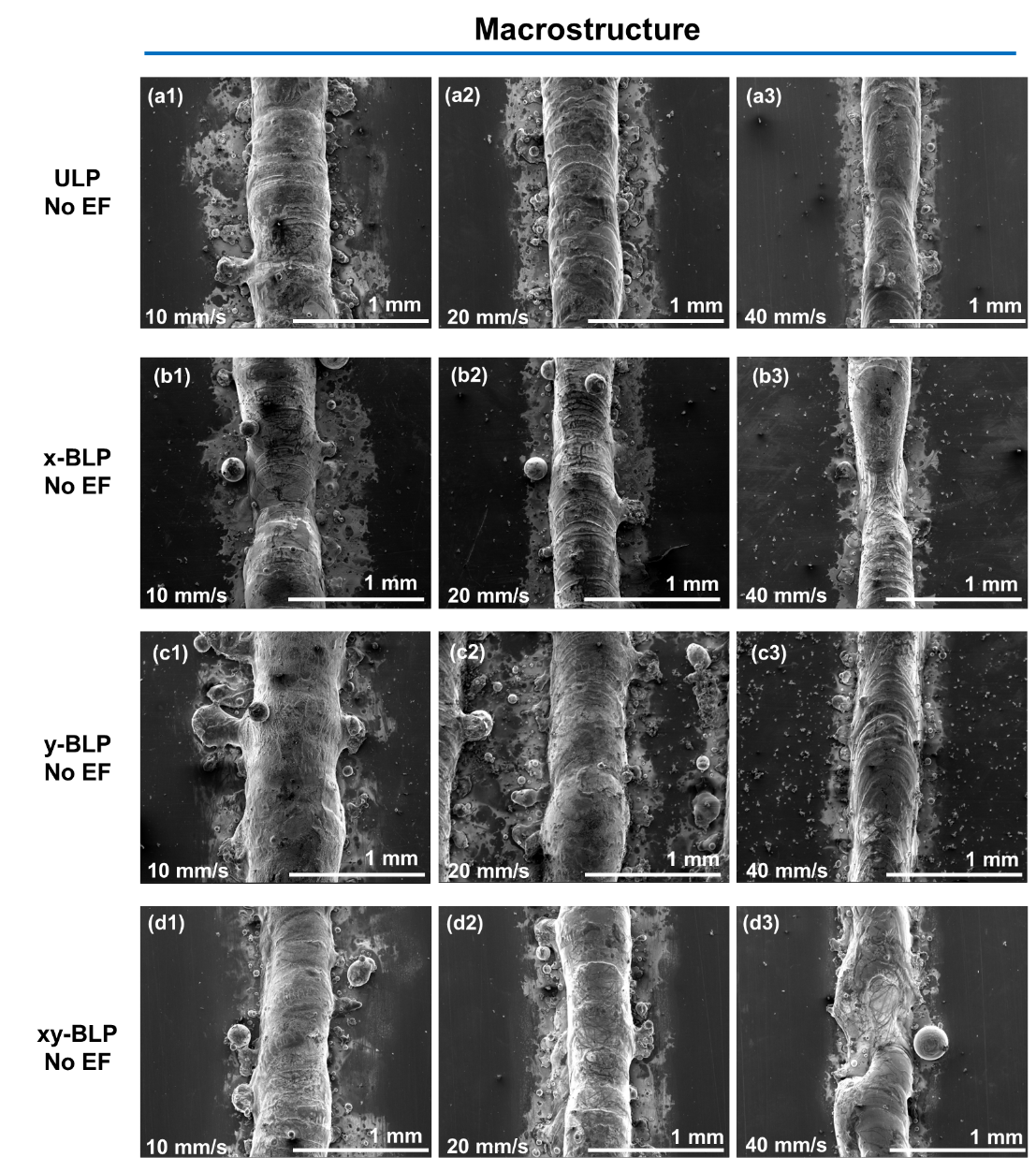
**

**Figure** S3: SEM images of the solidified SS316L melt tracks (at various speeds). ULP parameters: single laser power: 200 W, and BLP parameters: two lasers of 100 W each.


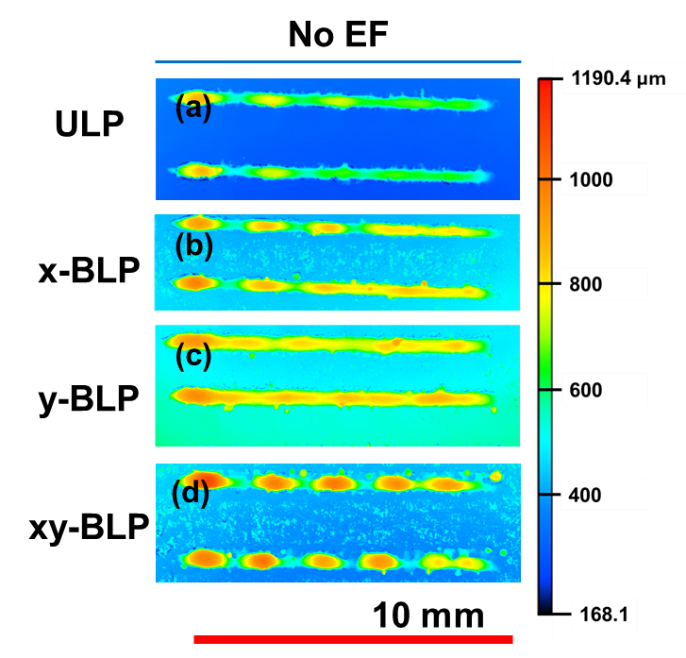


**Figure** S4: Confocal images of the solidified SS316L melt tracks (at constant speed of 40 mm/s). ULP parameters: single laser power: 200 W, and BLP parameters: two lasers of 100 W each.

1. **Assessment parameters for macrostructural analysis of xy-BLP process**

The trends in enhancing and deteriorating undulation stability have been observed through another parameter $\left( \frac{Z_{avg}}{Z_{max}} \right)$ (**Figure** S5). $\left( \frac{Z_{min}}{Z_{max}} \right)$ interprets as the average of the crests and troughs (z height) of the molten track that are closer to the crests show better stability of melt tracks. The trends for $\left( \frac{Z_{avg}}{Z_{max}} \right)$ are fairly in line with the $\left( \frac{Z_{min}}{Z_{max}} \right)$ parameter for xy-BLP with both DC and AC EFs. Other assessment parameters such as standard deviation and coefficient of variation are investigated to observe the effects of EF on the melt track instability. The mentioned parameters are determined from the Z_avg_. Therefore, in order to obtain a more stable melt track (reduced undulations), a lower standard deviation and coefficient of variation is preferred as observed in **Figure** S5. Parallel DC EFs overall show a low standard of deviation and coefficient of variation values when compared to that of its control counterpart (**Figure** S5(c,e)). Moreover, AC EFs with f < 100 kHz or f > 5 kHz also show values lower than the control, indicating stable melt tracks. Whereas for frequencies between 100 Hz ≤ f ≤ 5 kHz shows higher standard deviation and coefficient of variation values than the control, indicating enhanced undulations in the melt track (**Figure** S5(d,f)).


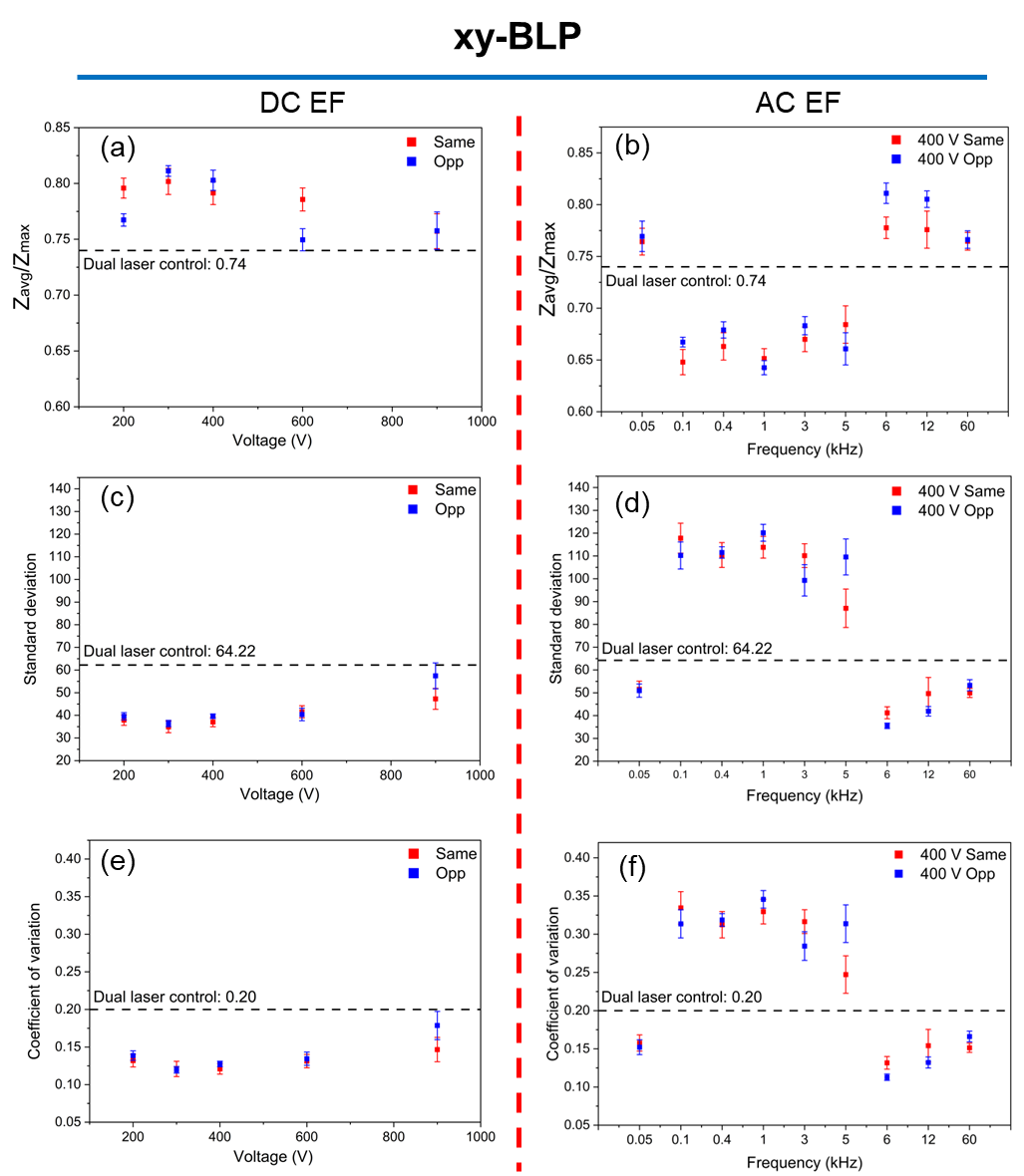


**Figure** S5: Assessment parameter for parallel (a,c,e) DC EF, (b,d,f) AC EF. (a) $\left( \frac{Z_{avg}}{Z_{max}} \right)$, (c) standard deviation, (e) coefficient of variation with increasing voltage (for DC EF). (b) $\left( \frac{Z_{avg}}{Z_{max}} \right)$, (d) standard deviation, (f) coefficient of variation with increasing frequency at various voltages (for AC EF).


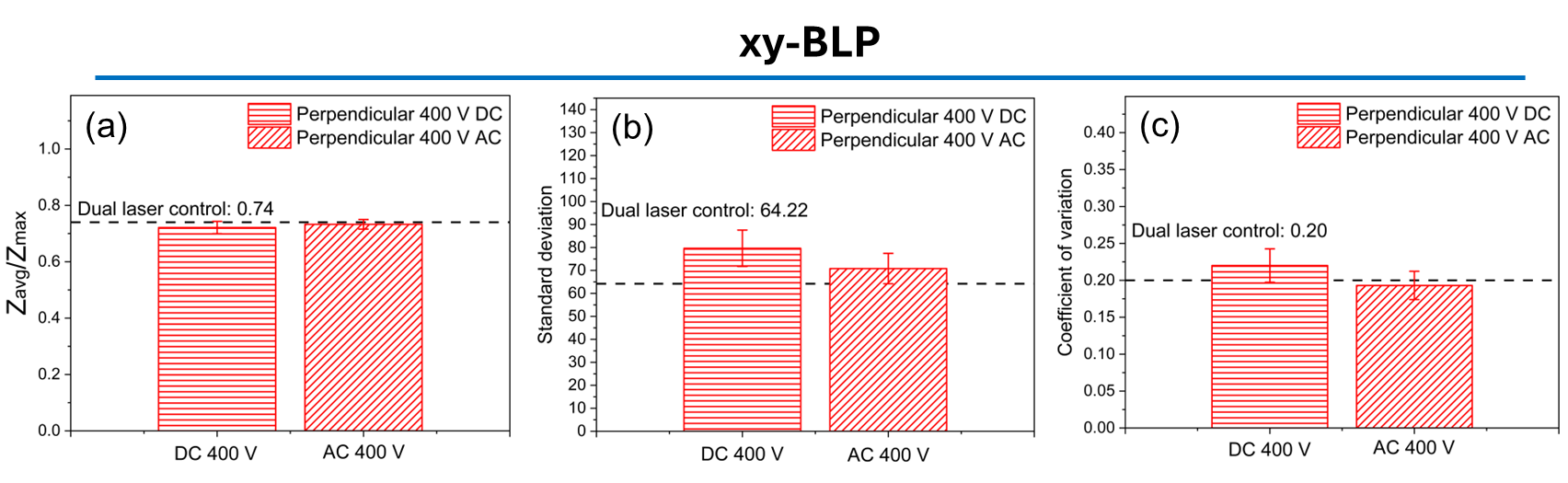


**Figure** S6: Assessment parameter for perpendicularly applied DC (400 V) and AC (400 V, 6 kHz) EFs. (a) $\left( \frac{Z_{avg}}{Z_{max}} \right)$, (b) standard deviation, (c) coefficient of variation.

1. **Solidification microstructure and analysis**


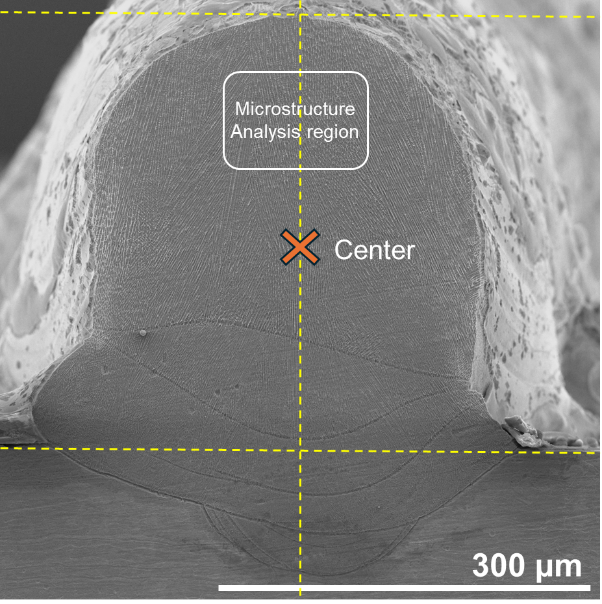


**Figure** S7: Illustration of the region for microstructural analysis.


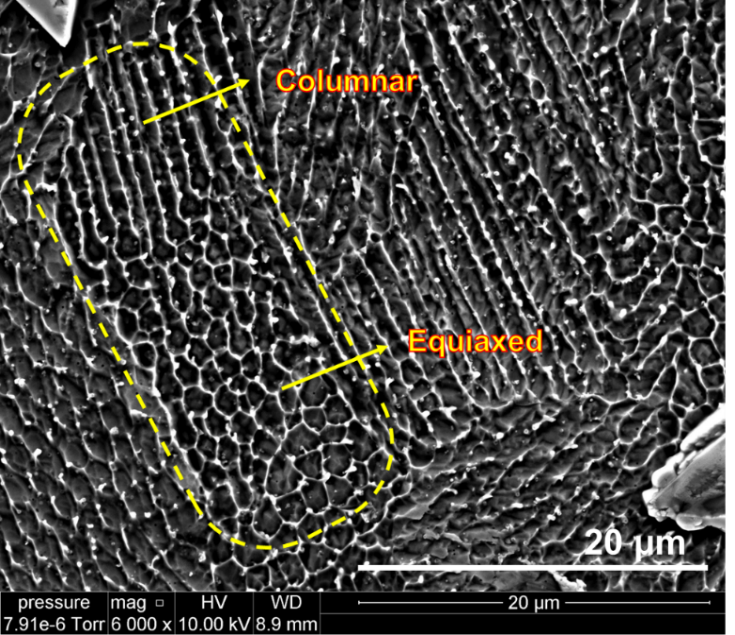


**Figure** S8: Illustration of different solidification structures present on SS316L.


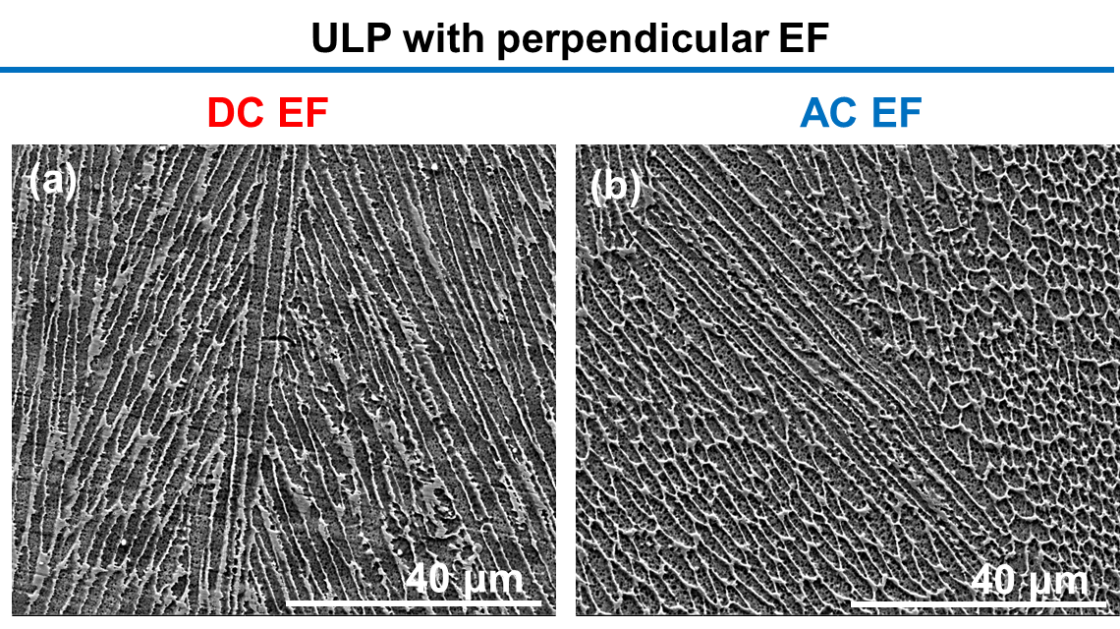


**Figure** S9: ULP microstructures formed under the influence of perpendicular EFs (c) DC (400 V) and (d) AC (400 V, 6 kHz).

**Table**  S1: Distribution of (a) grain area and (b) grain aspect ratio obtained for the various xy-BLP and ULP processes investigated.

| **Normal** | | | | |
| --- | --- | --- | --- | --- |
|  | **Size (μm^2^)** | | **Aspect ratio** | |
|  | **μ** | **σ** | **μ** | **σ** |
| **400V DC parallel xy-BLP** | 4.998 | 17.769 | 2.297 | 1.007 |
| **400V AC parallel xy-BLP** | 3.074 | 2.948 | 2.689 | 1.763 |
| **Control xy-BLP** | 8.970 | 70.309 | 5.609 | 6.416 |
| **400V AC perpendicular xy-BLP** | 5.772 | 5.198 | 2.427 | 1.338 |
| **400V DC perpendicular xy-BLP** | 9.590 | 53.442 | 2.542 | 1.237 |
| **Control ULP** | 24.806 | 79.373 | 11.919 | 12.348 |
| **400V AC parallel ULP** | 15.920 | 30.634 | 3.343 | 1.690 |
| **400V DC parallel ULP** | 5.161 | 5.784 | 3.578 | 3.193 |

1. **Modeling electric fields and thermal characteristics in laser processing**

The electrostatics module is utilized to incorporate the physics using Eqs. (S1) and (S2). Where the dielectric model is defined using the dielectric permittivity ($D=\varepsilon_{0}\varepsilon_{r}E$), expanding **Equation**  (S2) to **Equation** (S3). Where $\varepsilon_{0}$ is the permittivity of free space (8.85 × 10^-12^ m^-3^ kg^-1^ s^4^ A^2^) and ε_r_ is the relative permittivity. For simplicity, the particles are assumed to be spherical. As notable oxygen signals were observed for the surfaces of the SS316L powder used in this research (**Figure** S10), it was assumed that the powders possessed an oxide layer of Fe_2_O_3_ ^5-7^. The properties for calculation were utilized as present in COMSOL material library and the relative permittivity (ε_r_) according to ref. ^8^. Other temperature dependent properties (for SS316L) such as density (ρ) and surface tension (γ) are derived for refs. ^9^ and ^10^ respectively.

$E=-\nabla V$ (S1)

$\nabla.D=\rho_{v}$ (S2)

$\nabla.\left( \varepsilon_{0}\varepsilon_{r}E \right)=\rho_{v}$ (S3)


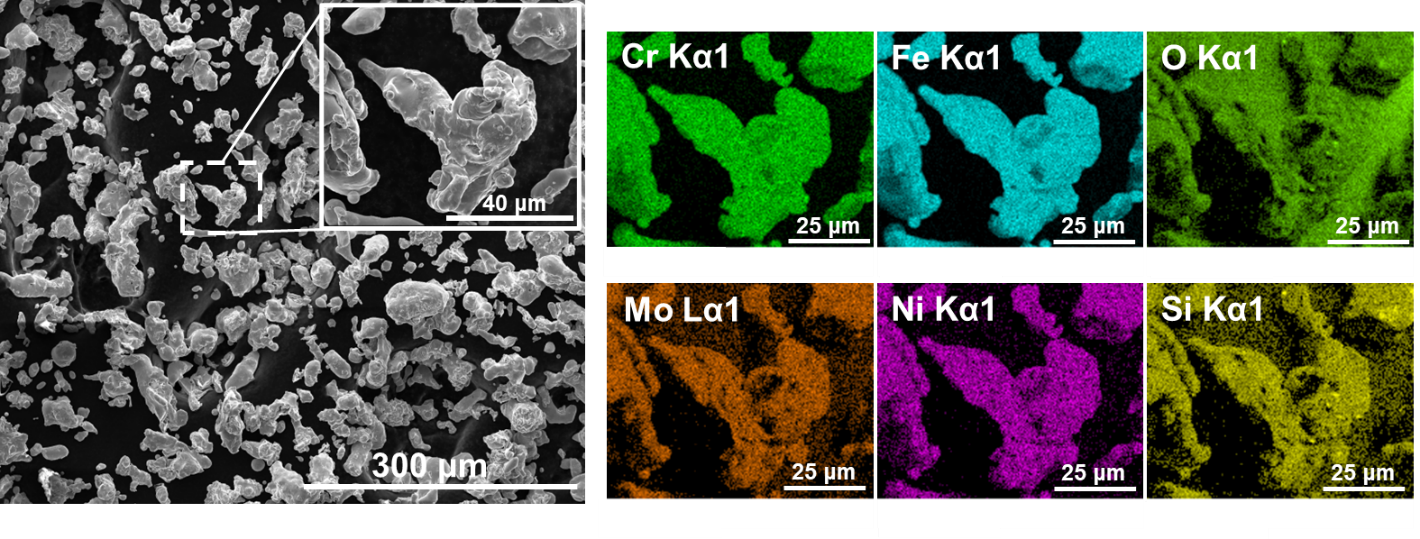


**Figure** S10: EDS spectral maps of the SS 316L powder used in this research.

According to electrical resistance tests, the SS316L powder bed utilized in this work is non-conductive. Energy-dispersive spectroscopy (EDS) elemental maps (**Figure** S10) revealed an oxide layer insulating individual particles, preventing electrical conduction and thereby enabling EF induction within the bed. Based on this oxide layer, the magnitude and transient behavior of these fields were estimated through finite element method (FEM) simulations in COMSOL. Under a potential of 400 V, maximum EF values reached 3.46 × 10^4^ V m^-1^ for DC and 4.27 × 10^4^ V m^-1^ for AC (**Figure** S11(a,b)). Transient FEM analyses at probe sites adjacent to individual particles (**Figures** S11(c,d)) further showed that EF magnitude scales directly with applied potential in the range of 200–900 V. During laser irradiation, local melting forms a pool surrounded by non-conductive SS316L powders, leading to fluid deformation through surface-tension effects ^11^. Furthermore, the nonuniform and random shape and distribution of the powders generate a diverse range of EFs, leading to complex multi-physics interactions that collectively drive the behavior observed in this work.


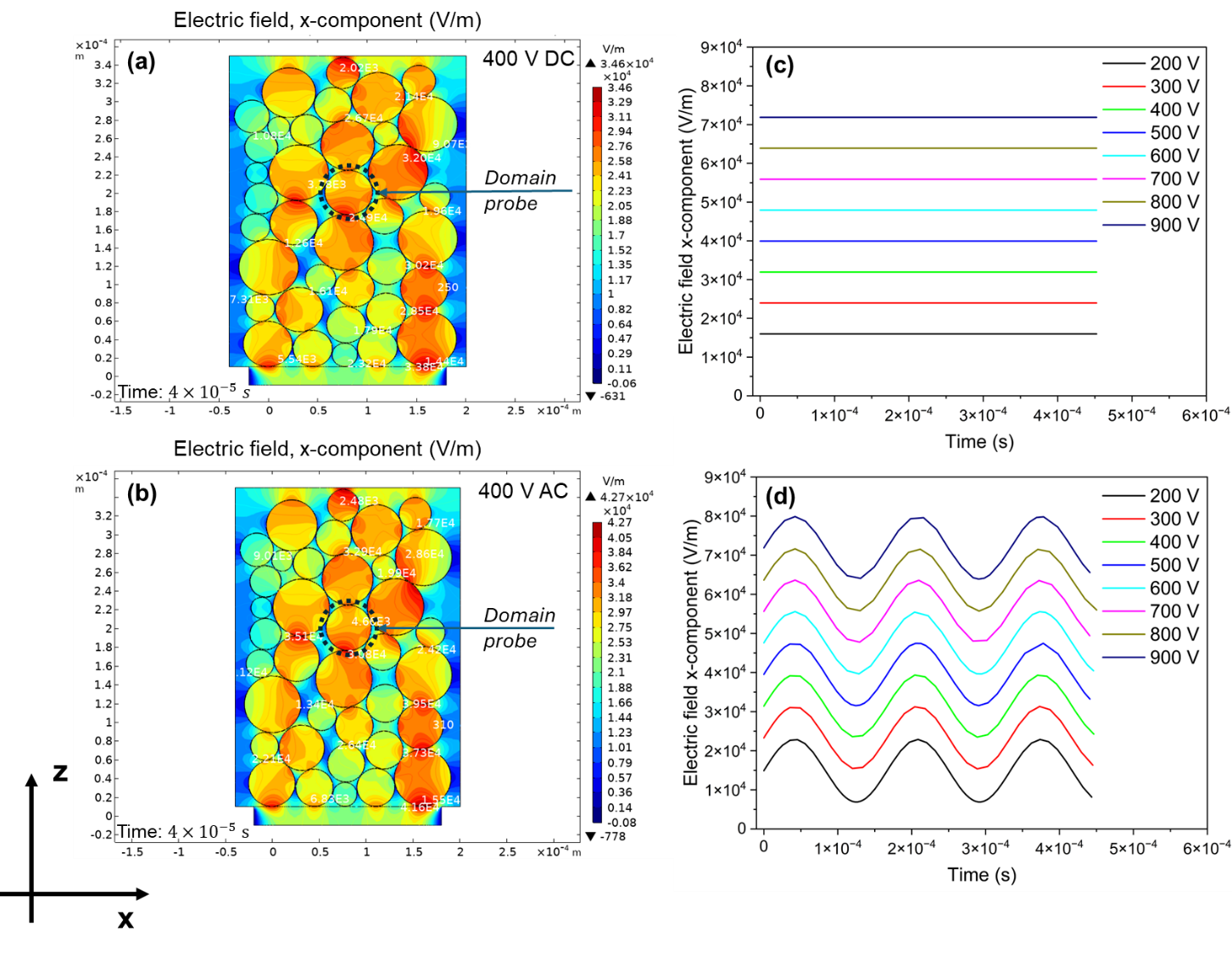


**Figure** S11: EF map of a dummy powder bed with a 400 V (a) DC and (b) AC potential. Transient maximum EF magnitudes at the edge of the particles highlighted in (a,b) for various applied (c) DC and (d) AC potentials.

In addition to the above, an IR camera was used to check for changes in the powder bed temperature upon the application of an EF without any laser processing. For all temperature measurements, a correction faction was used as given by ref^12^ as $T_{real}=\frac{T_{measured}}{\sqrt[4]{\cos\theta}}$. Where, $T_{real}$ is the real temperature which is reported and compared to FEM simulations for validations, $T_{measured}$ is the temperature obtained from the camera, $\sqrt[4]{\cos\theta}$ is the correction factor, and $\theta$(= 45°) is the angle at which the camera is positioned with respect to the powder bed horizontal. The temperature of the powder bed was recorded to bed 299.15 K without the application of an EF as shown in **Figure** S12. Subsequently, a DC and AC EF was applied and the temperatures of the powder bed after 5 mins were recorded, and found to be 299.37 K and 299.48 K respectively. Therefore, little to almost no changes in temperature was observed upon the application of EFs.


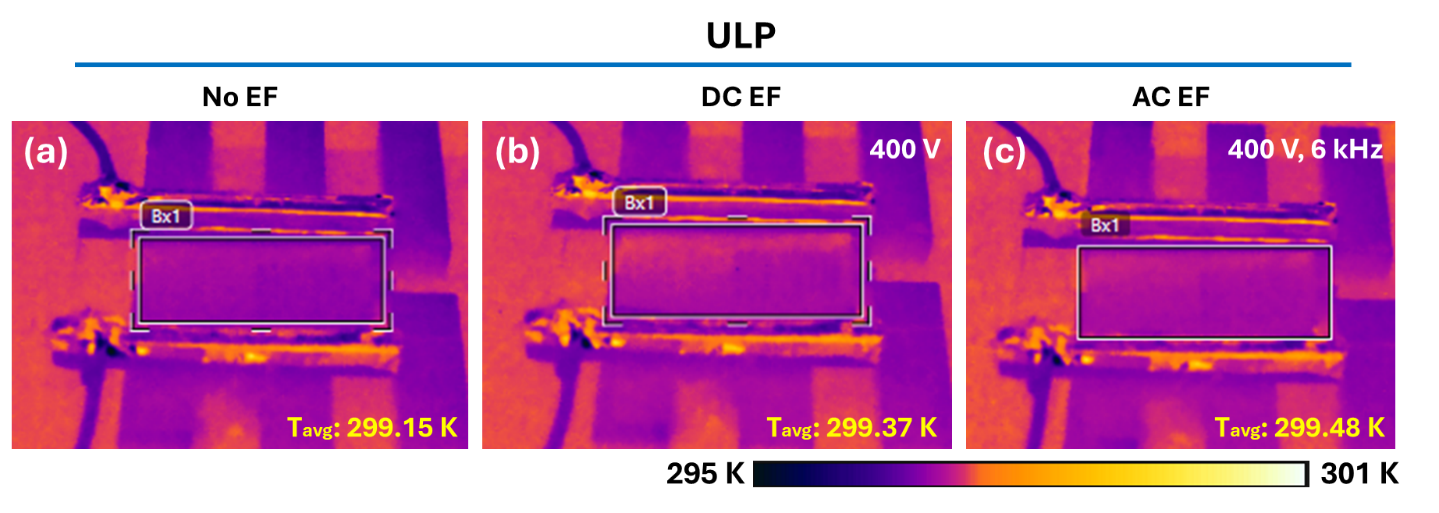


**Figure** S12: IR thermal images of the powder bed with and without EFs. (a) no EF, (b) DC EF (400 V), and (c) AC EF (400 V, 6 kHz).

A three-dimensional numerical simulation was conducted using COMSOL to model the thermal field during laser irradiation. Important thermophysical, material, and geometrical properties are deﬁned under global deﬁnitions. New material is deﬁned in COMSOL for introducing temperature-dependent properties of SS316L. Various thermophysical properties were utilized from past reference ^9^ and are illustrated in **Figure** 13(b). Subsequently, the heat transfer module is utilized for temperature calculations. Suitable edges are deﬁned for laser input, convective cooling, and surface to ambient radiation. Subsequently, for accurate thermal estimations, proper choice of heat sources is essential^3^ and for this work, two Gaussian beams with equal beam diameters are utilized. The energy input for the laser beams are defined using a gaussian function ^13^:

$Q\left( x,y,z \right)=P\left( 1-R_{c} \right)\frac{A_{c}}{\pi\sigma_{x}\sigma_{y}}e^{-\left[ \frac{\left( x-x_{0} \right)^{2}}{2\sigma_{x}^{2}}+\frac{\left( y-y_{0} \right)^{2}}{2\sigma_{y}^{2}} \right]}.e^{-A_{c}z}$ (S4)

Where P is the total power, $R_{c}$ is the reflection coefficient (Absorptivity ~0.7)^14^, $A_{c}$ is the absorption coefficient (experimentally determined), $\sigma_{x}$ and $\sigma_{y}$ are the x and y deviations of the laser beam, $e^{-\left[ \frac{\left( x-x_{0} \right)^{2}}{2\sigma_{x}^{2}}+\frac{\left( y-y_{0} \right)^{2}}{2\sigma_{y}^{2}} \right]}$ denotes the gaussian distribution in xy-plane and $e^{-A_{c}z}$ is the exponential decay due to absorption. x and y are the instantaneous coordinates defined by a straight line (**Equation** (S5) and (S6)) depending on the motion of the two lasers. $x_{0}$ and $y_{0}$ are the reference/origin coordinates.

The leading laser defined through a straight line is as follows:

$x=x_{0}+vt$ and $y=y_{0}$ (S5)

The trailing laser is defined with suitable x- and y-offsets as follows:

$x=x_{0}+x_{offset}+vt$ and $y=y_{0}+y_{offset}$ (S6)

Boundary conditions are set through heat equations,
$\dot{q}=-k\nabla T$ , $\dot{q}=h\Delta T$ and $\dot{q}=\sigma eA\left( T^{4}-T_{0}^{4} \right)$ (S7)

Where, k is the thermal conductivity, h is convective heat transfer coefficient, $\sigma$ is the Stefan Boltzmann constant, and e is the emissivity. The transient maximum temperatures are extracted for the entire geometry and at locations as shown in **Figure** 13(a). The meshing is divided into two sections. The center domain where the laser is scanned is finely meshed, and the domain around the center which has coarse mesh as shown in **Figure** S13(a)(inset). In total the meshing consists of 713559 vertices, 4241642 tetrahedra, 70862, 1116 edge elements, 16 vertex elements. Furthermore, the thermophysical properties are plotted as per COMSOL material library and ref. ^9^ as shown in **Figure** 13(b).

| 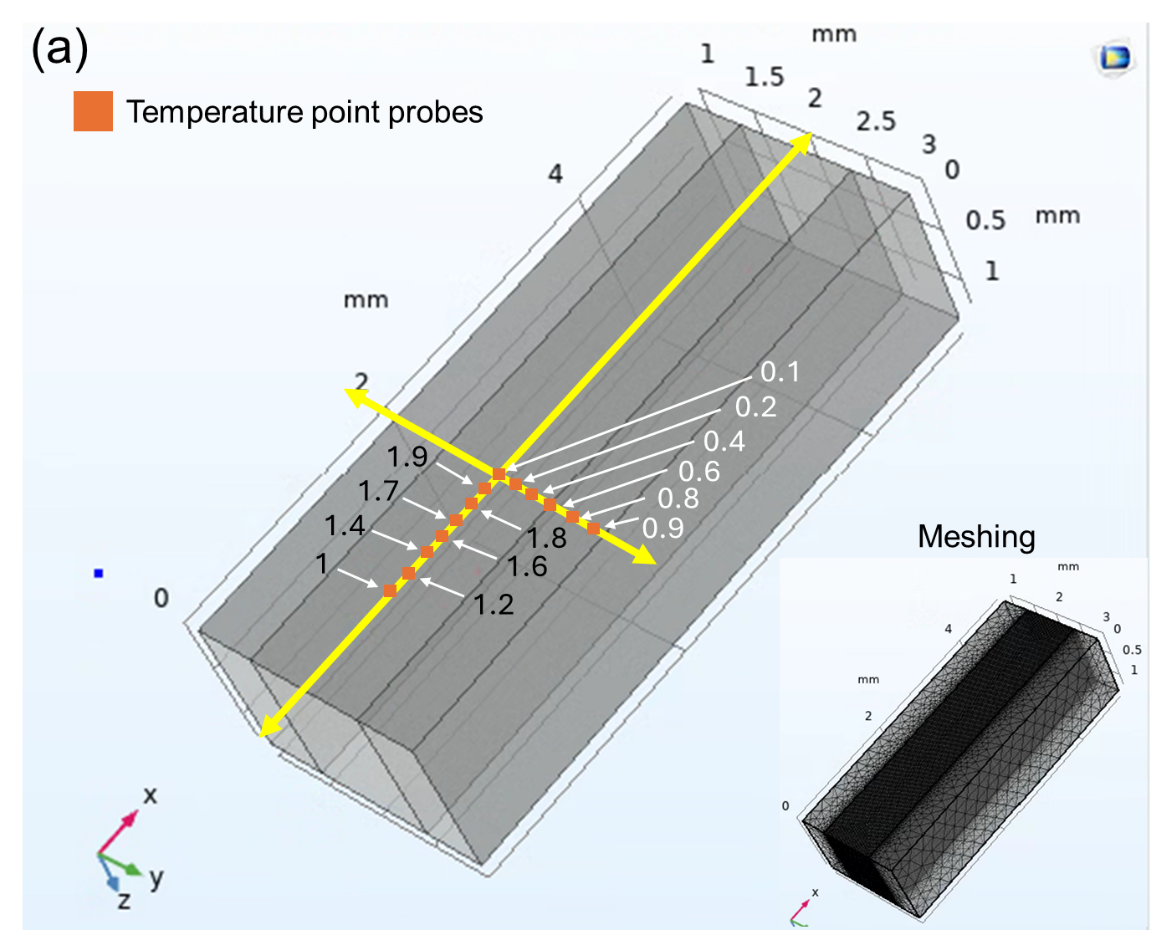 |
| --- |
| 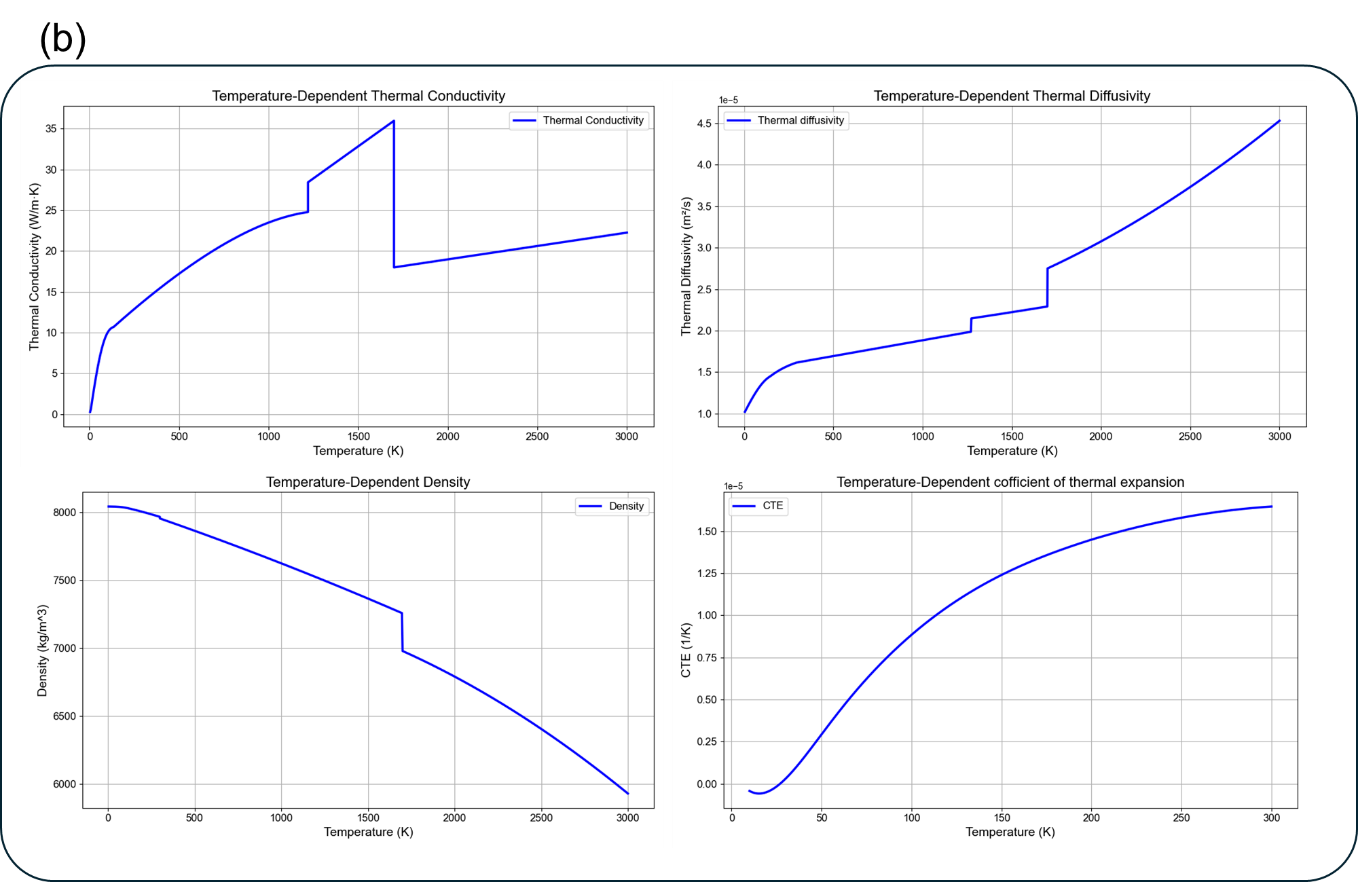 |

**Figure S13**: (a) Illustration of the domain probes located on the x- and y-directions with transient temperature extraction. Inset: Illustration of the meshing utilized for the FEM ananlysis. (b) Thermophysical properties utilized in this work as per stainless steel 316L COMSOL material library and Ref. ^9^

The popular FEM thermal model is validated for the maximum temperature values with IR thermography (FLIR, T865). The camera has a higher range of 300-2000 °C or 573-2273 K, and considering the melting temperature of SS316L at 1700 K, only a short window is available (1700-2273 K). Considering this lower laser power of 140 W was chosen, ensuring to melt the powder bed at the same scan speed as used in the experiments (40 mms^-1^). The emissivity for the utilized experiments used emissitivity of 0.25-0.3^15,16^. **Figure** S14(a) shows the IR thermograph of an experiment conducted with aforementioned laser paramters of 140 W and 40 mms^-1^ and were repeated 4 times to report the average with a deviation as seen in **Figure** S14(b).


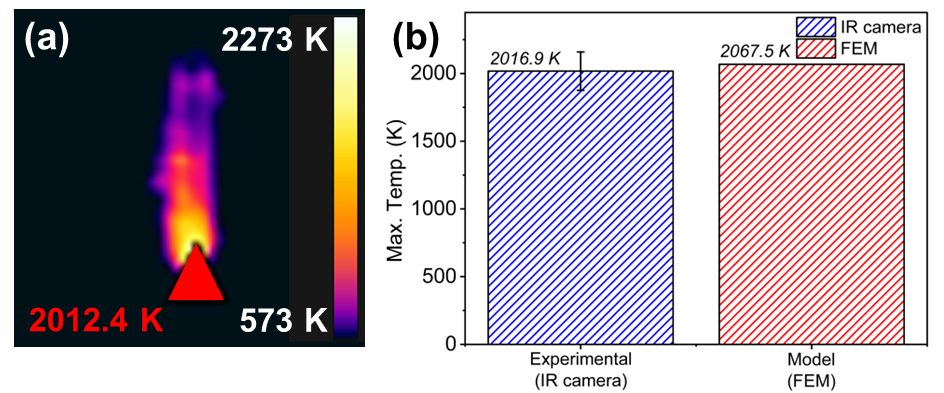


**Figure** S14: Validating experimentalto FEM model. (a) experimental temperation dermination using IR camera, and (b) Experimental and FEM model based determination of maximum temperature (under laser powder of 140 W, scan speed of 40 mms^-1^).

1. **Additional chemical characterizations**

**Figure** S15 shows the Raman spectra of various processesd and unprocessed samples. Wavenumber of ~478 corresponds to: α-FeOOH ^17^, maghemite (γ-Fe_2_O) ^18^. Higher wavenumber bands (>1000 cm^-1^) are mostly due to contaminations except for at 1440 cm^-1^ which corresponds to maghemite ^19^. FeO and Fe_3_O_4_ demonstrate weak spectra compared to Fe_2_O, α-FeOOH, and γ-FeOOH having strong spectra ^17^.





**Figure** S15: Raman spectra of xy-BLP polished cross-sections.

XPS results in **Figure** S16 enable visualization of the oxidation peaks of the SS316L post LPBF process ^20,21^. Low binding energy peaks 200 eV and less can be ignored as the arise mostly form organic contamination. Mo peaks occur around 225 eV. Subsequently traces of Carbon peaks are seen at binding energies of 275 eV. Enhanced oxidation peaks observed for the LPBF samples compared to the base plate are due to the thermal nature of the process made under ambient conditions.


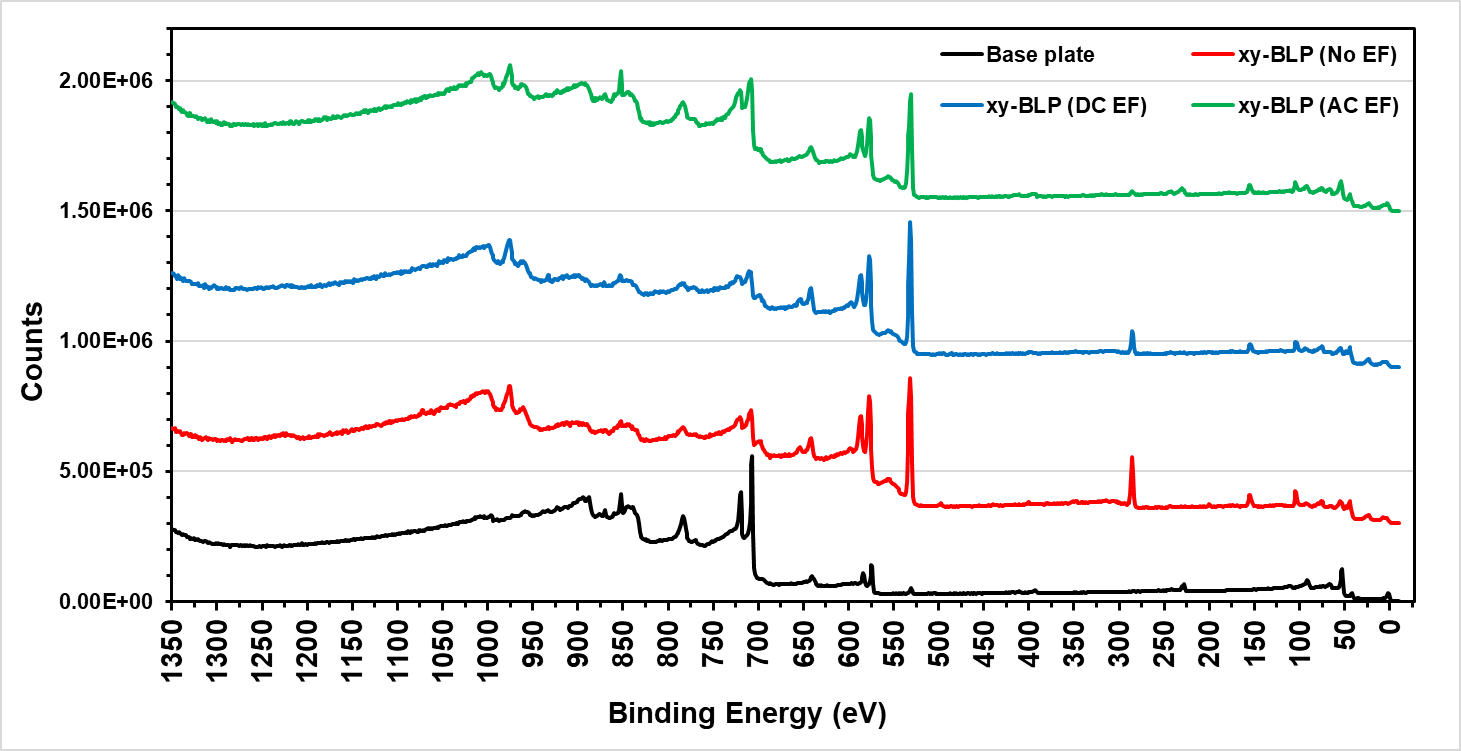


**Figure** S16: XPS spectra of base plate and xy-BLP samples.

**References**

1 Choo, H. *et al.* Effect of laser power on defect, texture, and microstructure of a laser powder bed fusion processed 316L stainless steel. *Materials & Design* **164**, 107534 (2019).

2 Amiri, M. & Payton, E. An analytical model for prediction of denudation zone width in laser powder bed fusion additive manufacturing. *Additive Manufacturing* **48**, 102461 (2021).

3 Zhang, W., Hou, W., Deike, L. & Arnold, C. B. Using a dual-laser system to create periodic coalescence in laser powder bed fusion. *Acta Materialia* **201**, 14-22 (2020).

4 Du, Y. & Arnold, C. B. Powder melting efficiency during laser powder bed fusion of stainless steel and titanium alloy. *Journal of Manufacturing Processes* **120**, 161-169 (2024).

5 Hwang, Y.-J., Wi, D.-Y., Kim, K.-S. & Lee, K.-A. High Temperature Oxidation Behavior of 316L Austenitic Stainless Steel Manufactured by Laser Powder Bed Fusion Process. *Journal of Powder Materials* **28**, 110-119 (2021).

6 Cheng, X., Feng, Z., Li, C., Dong, C. & Li, X. Investigation of oxide film formation on 316L stainless steel in high-temperature aqueous environments. *Electrochimica Acta* **56**, 5860-5865 (2011).

7 Yang, X., Gao, F., Tang, F., Hao, X. & Li, Z. Effect of surface oxides on the melting and solidification of 316L stainless steel powder for additive manufacturing. *Metallurgical and Materials Transactions A* **52**, 4518-4532 (2021).

8 Lunt, R. A., Jackson, A. J. & Walsh, A. Dielectric response of Fe2O3 crystals and thin films. *Chemical physics letters* **586**, 67-69 (2013).

9 Kim, C. S. Thermophysical properties of stainless steels. (Argonne National Lab., Ill.(USA), 1975).

10 Pichler, P., Leitner, T., Kaschnitz, E., Rattenberger, J. & Pottlacher, G. Surface tension and thermal conductivity of NIST SRM 1155a (AISI 316L stainless steel). *International Journal of Thermophysics* **43**, 66 (2022).

11 Tonks, L. A theory of liquid surface rupture by a uniform electric field. *Physical Review* **48**, 562 (1935).

12 Vasilevskyi, O., Woods, A., Jones, M. & Cullinan, M. In situ monitoring and quality assessment of laser powder bed fusion process for 316L stainless steel. *The International Journal of Advanced Manufacturing Technology* **140**, 6005-6026 (2025).

13 Mishra, A. K., Aggarwal, A., Kumar, A. & Sinha, N. Identification of a suitable volumetric heat source for modelling of selective laser melting of Ti6Al4V powder using numerical and experimental validation approach. *The International Journal of Advanced Manufacturing Technology* **99**, 2257-2270 (2018).

14 Trapp, J., Rubenchik, A. M., Guss, G. & Matthews, M. J. In situ absorptivity measurements of metallic powders during laser powder-bed fusion additive manufacturing. *Applied Materials Today* **9**, 341-349 (2017).

15 Becker, T., Stark, T., Arduini, M., Manara, J. & Altenburg, S. J. Knowing the spectral directional emissivity of 316L and AlSi10Mg PBF-LB/M surfaces: gamechanger for quantitative in situ monitoring. *Progress in additive manufacturing* **9**, 695-704 (2024).

16 Terrazas-Nájera, C. A., Fernández, A., Felice, R. & Wicker, R. On the thermal emissive behavior of four common alloys processed via powder bed fusion additive manufacturing. *Additive Manufacturing* **82**, 104023 (2024).

17 Thibeau, R. J., Brown, C. W. & Heidersbach, R. H. Raman spectra of possible corrosion products of iron. *Applied spectroscopy* **32**, 532-535 (1978).

18 Chourpa, I. *et al.* Molecular composition of iron oxide nanoparticles, precursors for magnetic drug targeting, as characterized by confocal Raman microspectroscopy. *Analyst* **130**, 1395-1403 (2005).

19 Sousa, M. H., Tourinho, F. A. & Rubim, J. C. Use of Raman micro‐spectroscopy in the characterization of MIIFe2O4 (M= Fe, Zn) electric double layer ferrofluids. *Journal of Raman Spectroscopy* **31**, 185-191 (2000).

20 Choi, W. T., Oh, K., Singh, P. M., Breedveld, V. & Hess, D. W. Hydrophobicity and improved localized corrosion resistance of grain boundary etched stainless steel in chloride-containing environment. *Journal of The Electrochemical Society* **164**, C61 (2017).

21 Choi, W. T., Oh, K., Singh, P. M., Breedveld, V. & Hess, D. W. Wettability control of stainless steel surfaces via evolution of intrinsic grain structures. *Journal of Materials Science* **51**, 5196-5206 (2016).
